# A 25‐year climatology of low‐tropospheric temperature and humidity inversions for contrasting synoptic regimes at Neumayer Station, Antarctica

**DOI:** 10.1002/joc.7780

**Published:** 2022-07-10

**Authors:** Tiago Silva, Elisabeth Schlosser, Manuela Lehner

**Affiliations:** ^1^ Department of Geography and Regional Sciences University of Graz Graz Austria; ^2^ Department of Atmospheric and Cryospheric Sciences University of Innsbruck Innsbruck Austria; ^3^ Austrian Polar Research Institute Vienna Austria

**Keywords:** humidity inversions, radiosondes, synoptic conditions, temperature inversions, weather observations

## Abstract

A 25‐year set of daily radiosonde data was used to investigate temperature and humidity inversions at Neumayer Station, coastal Dronning Maud Land, Antarctica. For the first time, inversions were studied differentiating between different synoptic conditions and different height levels. It was shown that, generally, inversions occurred on the majority (78%) of the days, with simultaneous occurrence of humidity and temperature inversions being observed on approximately two thirds of all days. Multiple inversions are common in all seasons for cyclonic and noncyclonic conditions, however, typically occur more frequently under cyclonic conditions. The seasonality of inversion occurrence and features, that is, inversion strength, depth and vertical gradients, was analysed statistically. Different formation mechanisms depending on inversion levels and prevailing weather situations are related to typical annual courses of certain inversion features. Winter maxima were found for the features that are mostly connected to the temperature close to the surface, which is mainly a result of the negative energy balance, thus influencing surface‐based inversions. At the second level, both temperature and humidity inversions are often caused by advection of comparably warm and moist air masses related to the passage of cyclones and their frontal systems. Hence, maxima in several inversion features are found in spring and fall, when cyclonic activity is strongest. Monthly mean profiles of humidity and temperature inversions reveal that elevated inversions are often obscured in average profiles due to large variations in inversion height and depth.

## Graphical abstract

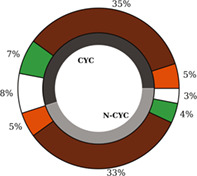

## 1 INTRODUCTION

Temperature and humidity inversions are a common feature of the stable boundary layer (SBL) in polar regions (e.g., Phillpot and Zillman, 1970; Serreze *et al*., 1992; King and Turner, 2007; Sedlar and Tjernström, 2009; Zhang *et al*., 2011; Nygård *et al*., 2013; Vignon *et al*., 2019). Basically, two types of inversions can be distinguished, surface‐based and elevated inversions. Surface‐based inversions in polar regions result from the negative surface energy balance, which is supported by the large solar zenith angle, the high albedo of snow‐ and ice‐covered surfaces, and the prolonged absence of solar radiation during the polar night (Andreas *et al*., 2000). Lack of turbulent mixing and thus reduced vertical transport of heat and moisture can lead to extreme inversion thickness and strength. Elevated inversions are often associated with horizontal advection of relatively warm and moist air masses from lower latitudes by synoptic scale processes (e.g., Shupe *et al*., 2013; Brunke *et al*., 2015).

Both humidity and temperature inversions have a strong influence on the surface energy balance and thus also on the mass balance of glaciers and ice sheets. In particular, humidity inversions play an important role in the hydrological cycle, which is enhanced in cases of atmospheric rivers that lead to large amounts of precipitation, thus linking humidity inversions to the surface mass balance of the large ice sheets of Greenland and Antarctica and consequently to sea level changes (Gorodetskaya *et al*., 2020). In spite of the importance of humidity inversions for both the local meteorological conditions and the mass balance of high‐latitude ice sheets (King and Turner, 2007), they have received much less attention than temperature inversions and their formation mechanisms in the past.

A special application of inversion studies is the paleoclimatic interpretation of ice cores. The derivation of paleotemperatures from stable water isotopes is based on the relationship between stable isotope ratios of ice (i.e., former precipitation) and the condensation temperature, not the surface temperature. At most deep drilling locations, strong inversions prevail, which has to be taken into account for a correct interpretation of ice core data. The relationship between surface temperature, condensation temperature and temperature at the top of the inversion is still a fairly unknown subject; the latter has been used as approximation to the condensation temperature in ice core studies for many years (e.g., Stenni *et al*., 2016).

Generally, temperature inversions are better understood than humidity inversions, and the majority of studies covers the Arctic Ocean. Various studies employed radiosonde data and/or remote sensing techniques to study the lowest part of the troposphere in the Arctic (e.g., Kahl, 1990; Liu *et al*., 2006; Devasthale *et al*., 2011; Vihma *et al*., 2011) and Antarctic (e.g., Andreas *et al*., 2000; König‐Langlo and Loose, 2007; Tomasi *et al*., 2011; Nygård *et al*., 2013; Dufour *et al*., 2019; Vignon *et al*., 2019), or in both hemispheres (e.g., Pavelsky *et al*., 2011; Zhang *et al*., 2011). Most of them had restricted temporal coverage and did not distinguish between different weather conditions. In particular, the interaction of inversions and low‐level stratus clouds and the involved long‐wave radiation fluxes (e.g., Sedlar and Tjernström, 2009; Sedlar *et al*., 2012; Shupe *et al*., 2013; Sedlar and Tjernström, 2017) as well as the influence of and on sea ice (Andreas *et al*., 2000; Pavelsky *et al*., 2011; Palo *et al*., 2017) were subjects of various investigations. Inversions in the interior of Greenland (Shahi *et al*., 2020) are best comparable to those above the Antarctic ice sheet (e.g., Hirasawa *et al*., 2000; Hudson and Brandt, 2005; Pietroni *et al*., 2014), but observations are scarce for both regions. Whereas only few studies are available for Antarctic coastal stations (see below), a relative abundance of investigations for the Arctic Ocean in summer as well as in winter is found. Truong *et al*. (2020) studied the boundary layer over the Southern Ocean between Tasmania and the Antarctic coast using a cluster analysis of radiosonde data. They found one cluster located along the Antarctic coastline, which would correspond to Neumayer conditions.

Melting sea ice constrains the surface temperature to the freezing point, so an inversion associated with low stratus clouds is observed frequently in the Arctic (e.g., Shupe *et al*., 2013; Palo *et al*., 2017). In winter, the thickness of the sea ice influences the surface temperature and thus the strength of the inversion (Bintanja *et al*., 2011). The highly complex interaction between surface inversions, elevated inversions and low‐level clouds within or capped by the inversion was studied by Sedlar *et al*. (2012) and Sedlar and Tjernström (2009, 2017). Whereas in the Arctic Ocean long periods of rather uniform weather conditions with low stratus clouds and a surface inversion above the melting sea ice prevail in summer, at Antarctic coastal stations similar processes that involve low stratus clouds are of minor importance since the synoptic conditions are strongly influenced by passing cyclones in the circumpolar trough (see section 2.1) and weather conditions are thus highly variable. This is supported by Figure S1, Supporting Information, which shows the density of consecutive days with the same weather situation (discussed therein). During the passing of a cyclone, highly turbulent conditions are found, followed by an anticyclonic, quiet phase with low cloudiness. The complex relationships between inversions at different levels and between temperature and humidity inversions have not been thoroughly investigated so far.

For Neumayer, only one previous study is available that investigates temperature inversions. However, Neumayer radiosonde data are included in two other studies of Antarctic inversions. König‐Langlo and Loose (2007) found that surface‐based inversions are common at Neumayer with a maximum of occurrence in winter and a minimum in summer. Seventy‐five percent of all inversions exhibit a strength, that is, temperature difference between inversion base and top, of more than 1 K and values up to 25 K can be reached. However, their study mainly considered surface‐based inversions under anticyclonic conditions. Nygård *et al*. (2013) used data from 11 coastal Antarctic stations, including Neumayer, to create a 10‐year climatology of humidity inversions. They state that generally humidity inversions occur most frequently in winter and spring and roughly half of the inversions occur simultaneously with temperature inversions. In a recent study, Vignon *et al*. (2019) combined an 8‐year data set from nine Antarctic coastal stations including Neumayer to analyse the vertical structure of the lowest 3 km of the troposphere. They found fundamentally different conditions at the various stations depending on the topography, namely ice shelf stations, strongly katabatically influenced locations and stations in a complex orography. In particular, a katabatic regime has a strong influence on humidity due to sublimation processes. At Neumayer, this is of minor importance. They also assessed the ability of two reanalysis products, ERA‐Interim and ERA5, to represent inversions and generally the structure of the lower troposphere and found that even for state‐of‐the‐art atmospheric models, this remains a challenge.

Most recently, Gorodetskaya *et al*. (2020) presented a study of atmospheric rivers for Neumayer and Syowa Stations, both coastal Antarctica. Atmospheric river events mean strongly enhanced poleward moisture transport leading to extraordinarily high precipitation amounts. Gorodetskaya *et al*. (2020) showed that a strong humidity and temperature inversion is found through the mid‐troposphere for atmospheric rivers, linked to an intensified low‐level jet (LLJ).

In extremely cold environments, radiosonde measurements remain a challenge and error possibilities are large. For instance, humidity sensors often experience a dry‐bias caused by solar heating (e.g., Miloshevich *et al*., 2001; Gettelman *et al*., 2006; Rowe *et al*., 2008) or a moist‐bias resulting from condensation and icing on the sensor when exposed to supercooled water (Nash, 2015). The delayed response of the sensors in an ascending radiosonde can also lead to errors, particularly close to saturation and where large vertical gradients in temperature and humidity occur. However, Hudson *et al*. (2004) showed that radiosondes can yield reliable results when sensors are properly calibrated to ambient conditions before launch.

At the German Antarctic wintering base Neumayer, in coastal Dronning Maud Land, radiosonde measurements have been carried out daily since 1983. In this study, a 25‐year data set (1993–2017) was used in combination with the corresponding meteorological surface data (SYNOP) to study temperature and humidity inversions and their interrelations. We present the first comprehensive, long‐term analysis of temperature and humidity inversions at an Antarctic coastal station. Since formation mechanisms are different for surface‐based and elevated inversions as well as for different weather conditions, for the first time, an inversion climatology was created dependent on weather conditions, inversion base height and season by statistically analysing inversion frequency, depth, strength and vertical gradients. Additionally, the relationship between inversion features as well as between temperature and humidity inversions and between inversions at different levels was investigated.

## 2 DATA AND METHODS

### 2.1 Study period and location

To assess the quality of radiosonde products is generally a challenging task. Dufour *et al*. (2019) compared radiosonde data from various Antarctic stations to four reanalysis products, namely ERA‐Interim, NCEP‐CFSR, JRA 55 and MERRA 2.

For Neumayer Station, the vertically integrated moisture transport anomalies agree well with the four reanalyses products from 1993 onwards. Since the timing of good agreement was different for other stations, we conclude that it is not due to an improvement in the reanalyses' data, but hints at a higher quality of the radiosonde data. Thus, the 25‐year period 1993–2017 was chosen for the present study, which was initiated in 2018.

Neumayer Station was constructed in 1981 on the Ekström Ice Shelf in coastal Dronning Maud Land, East Antarctica. The first base was given up for technical reasons and substituted by Neumayer II (70°39′S, 8°15′W) in 1992; and in February 2009, Neumayer II was replaced by Neumayer III (70°40′S, 8°16′W, hereafter called “Neumayer”), 5 km south of the former base (Wesche *et al*., 2016). For our study, only data from Neumayer II and III were used.

At Neumayer, the sun stays permanently above the horizon from November 19th to January 24th (polar day) and permanently below the horizon from May 19th to July 27th (polar night) (König‐Langlo and Loose, 2007). Ekström Ice Shelf is one of the smaller ice shelves in Antarctica, stretching south for approximately 120 km and is bordered by ridges of ~500 m elevation to the east and west. The surrounding ocean is covered by sea ice for the largest part of the year, only between mid‐January and mid‐March the coast becomes ice‐free.

**FIGURE 1:**
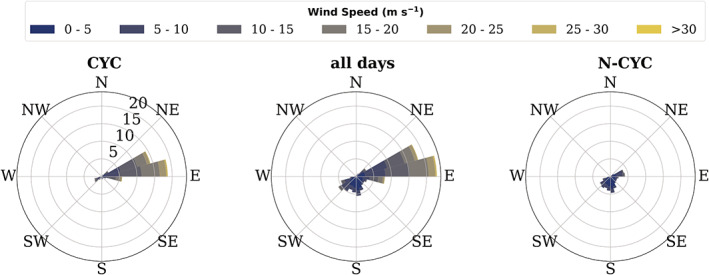
Observed wind speed and direction at Neumayer (wind rose) on days defined as cyclonic (a) and noncyclonic (c) conditions and for all days (b) [Colour figure can be viewed at wileyonlinelibrary.com]

Neumayer weather conditions are strongly determined by cyclone activity in the circumpolar trough. The weather is characterized by cyclones passing from west to east with the general westerly flow, with anticyclonic conditions for shorter or longer periods between two cyclones. The semi‐annual oscillation of the circumpolar trough corresponds to distinct surface pressure minima in spring and fall related to strong cyclone activity. Similarly, it can lead to longer anticyclonic periods in summer and winter, when the trough and thus the position of the frontal zone moves northwards. This is also indicated by the main wind directions (Figure 1). For the majority of the time Neumayer Station experiences relatively strong easterly to east‐northeasterly winds, related to the clockwise rotation of the passing cyclones. The centre of the cyclones is always north of the coast since the topography (increasing elevation) blocks further southward movements of the low‐pressure systems. Weaker winds from southerly or southwesterly directions prevail under high pressure (see also Klöwer *et al*., 2013) (section 2.4).

### 2.2 Synoptic weather observations

Since March 1981, SYNOP observations have been carried out at Neumayer every 3 hours between 0000 and 2100 UTC. At night (0300 and 0600 UTC), the observations are performed only automatically; thus, no visual observations (weather, clouds, visibility) are included. Measurements of surface pressure, temperature (at 2 m), dew‐point temperature (at 2 m) and wind vector (at 10 m height) are provided.

These data are coded in FM12‐SYNOP format and directly transferred to the Global Telecommunication Service (GTS) under the World Meteorological Organization (WMO) code‐number 89002, where it contributes to global weather forecasting models (König‐Langlo and Loose, 2007). The 25‐year series of SYNOP data are available at the Baseline Surface Radiation Network (BSRN), which collects and archives high‐quality ground‐based radiation measurements (Driemel *et al*., 2018). The 1200 UTC SYNOP data were used to classify inversions depending on the general weather condition (see section 2.4).

### 2.3 Upper air soundings

At Neumayer Station, radiosonde data are available since 1983. Usually one balloon ascent is performed per day measuring atmospheric profiles of air pressure, temperature, dew point temperature and wind vector at high resolution (König‐Langlo and Loose, 2007). Generally, the balloon is launched in the morning, so that the data are available for the GTS at 1200 UTC. Deviations from this time can occur due to problems with the start because of high wind speeds or a too early bursting of the balloon. In those cases, sometimes a second or even third balloon launch was carried out at a later time.

Different from the general requirements of the WMO for radiosonde data (5 pressure levels between the surface and the 500‐hPa level) (Nash, 2015), for the present study, we require more than 10 pressure levels with the surface level having a pressure higher than 925 hPa, since at Neumayer, the surface pressure is below 1,000 hPa (WMO standard) most of the time. Thus, here we use the radiosonde data set from BSRN (Driemel *et al*., 2018), which has a higher vertical resolution than the IGRA (Integrated Global Radiosonde Archive) data set (Durre *et al*., 2006), the latter providing mostly mandatory pressure levels. Following these criteria described above, a total of 8,489 observations is available, which corresponds to 93% of the days in the study period.

**FIGURE 2:**
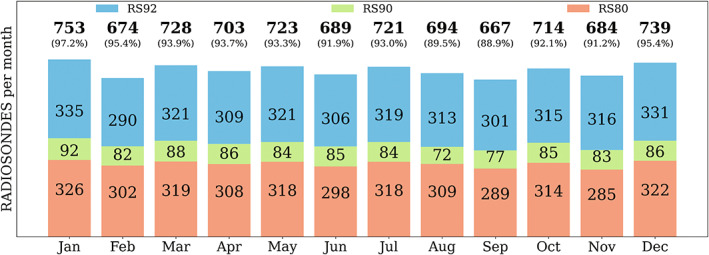
Total monthly radiosonde availability (bold numbers) from Baseline Surface Radiation Network under the stipulated criteria; the total monthly radiosonde availability depending on the radiosonde type used is indicated by the coloured bar portions [Colour figure can be viewed at wileyonlinelibrary.com]

Figure 2 presents the total monthly sum of observations at Neumayer used in this study. The background colour of each bar portion symbolizes the type of radiosonde device used. During the 25‐year period, three types of radiosondes were launched. Until late 2003, Vaisala RS80 (red) was used, followed by Vaisala RS90 (green) until late 2006, for the remainder of the study period Vaisala RS92 (blue) was utilized. There is only one gap in the data for the entire month of November 2003. The number of successful launches is only slightly lower in autumn and spring than in the other seasons, potentially as a consequence of severe weather conditions (blizzards). Thus, it is assumed that each month and season is similarly represented in the data set.

It has to be kept in mind that these data come from different sensors throughout the years. Not only the hardware but also the software has been gradually improved. Various error possibilities exist, starting with the storage of the sondes. In extremely cold and dry environments, such as Antarctica, radiosonde storage and launch at near ambient temperatures are known to minimize errors due to thermal shock (Hudson *et al*., 2004). The current and typical launch procedure includes a period of temperature adjustment before launch, which allows the radiosonde to balance with the surface temperature.

For the early Vaisala RS80, wind profiles were tracked by receiving OMEGA terrestrial navigational signals with an antenna (Maturilli and Kayser, 2017). Since the late nineties, wind speed and direction are monitored via balloon motion tracking.

Some radiosonde humidity sensors experience a dry‐bias caused by solar heating and a moist‐bias error (e.g., Miloshevich *et al*., 2006; Rowe *et al*., 2008), which may result from condensation and icing on the sensor when exposed to supercooled liquid water. From Vaisala RS80 to RS92, radiosondes at low temperatures in general are unable to respond quickly to the given sudden temperature change and the rapid decrease of water vapour near the surface, leading to a potential time‐lag at the first measured pressure levels (e.g., Miloshevich *et al*., 2001; Wang *et al*., 2002; Miloshevich *et al*., 2004; Tomasi *et al*., 2011). For RS80, Miloshevich *et al*. (2001) estimated that maximum time‐lag errors for relative humidity (RH) vary with temperature from about ±5% RH at −20°C to ±15% RH at −40°C. Such issues allowed the RS80‐humidity sensor to form an ice coating on the sensor in liquid‐water or ice‐saturated conditions, which sometimes caused the measurement to remain near ice‐saturation over large parts of the troposphere, not resembling the conditions of the ambient air (Maturilli and Kayser, 2017). Hudson *et al*. (2004) showed, however, that Vaisala RS80 radiosondes can measure temperature, pressure and humidity accurately in Antarctica when sensors are properly calibrated to ambient conditions before launch.

In comparison to RS80, RS90 response is known to be much faster with an estimated time lag of 2 s at −20°C (Miloshevich *et al*., 2004). For Vaisala RS90, as the humidity sensors are not shielded against solar radiation, incoming short‐wave radiation can heat the sensors during daytime launches, causing the so‐called daytime radiation dry bias. Currently, there is no proper correction for issues with sensor icing, and therefore they were not considered either. Furthermore, the two earlier types (RS80 and RS90) were tested rather inland under a more extreme environment compared to the near‐shore conditions. Since there are no studies assessing the associated impacts in accuracy of these sensors at the Antarctic margin, it is therefore assumed that some bias may still occur, but minimized.

According to Nash *et al*. (2011), the typical temperature uncertainty for RS92 within the lower troposphere is below 0.1 K. For typical temperature ranges at Neumayer, between 0 and −40°C, relative humidity errors are often below 1.5%. However, for values of relative humidity between 60 and 80%, when the air temperature is close to −50°C, errors can reach 2% during day and oscillate between −2 and 2 percentage points during night. The uncertainty associated with air pressure is always lower than 0.05 hPa regardless of the environmental conditions.

### 2.4 Definition of weather condition categories

As mentioned in section 2.1, different from the Arctic, weather and climate at Antarctic coastal stations are strongly influenced by the circumpolar trough, a climatological low‐pressure area that results from a number of cyclones that regularly develop and move eastwards above the Polar Ocean. Weather at Neumayer thus has a fairly “binary” character: either overcast conditions with precipitation and/or blowing/drifting snow and high wind speeds from easterly to north‐northeasterly directions related to a cyclone passing in the north of the base, or, between two cyclones, fair weather conditions with south to southwesterly winds and low cloudiness (e.g., König‐Langlo *et al*., 1998; König‐Langlo and Loose, 2007) (see also Figure S2, which shows a median cloudiness of 8 oktas, that is, overcast skies under cyclonic conditions and considerably lower cloudiness for noncyclonic conditions). Figure 1 shows the mean wind speed and direction at Neumayer observed on days defined as cyclonic (a) and noncyclonic (c) conditions and for all days (b). Note that, similar to König‐Langlo and Loose (2007), here we define “cyclonic” not as related to positive vorticity, but simply as “associated with a cyclone.” It is striking that the sector from southwest to northeast practically does not occur, whereas the east‐northeast‐east sector is overwhelmingly dominant. Also, the weak winds from southerly directions for noncyclonic conditions are clearly shown. Therefore, we also chose a rather simple, binary definition of weather conditions in order to take into account that mechanisms forming temperature and humidity inversions depend on the weather situation: the radiosonde observations were grouped into “cyclonic” and “noncyclonic” conditions using the SYNOP code of present (ww) and past weather (WW) based on Manual WMO Codes (2016). Basically, cyclonic conditions were defined as observations of significant precipitation or drifting/blowing snow at the time of the observation or in the preceding hour. Noncyclonic conditions include observations without any significant weather or with fog or diamond dust precipitation, that is, extremely small ice crystals that form under clear‐sky (or high‐cloud) conditions without cyclonic influence due to radiative cooling of the air (Curry, 1983). Both fog and diamond dust usually occur under quiet conditions with low wind speeds, thus they are included in the noncyclonic category. If the past weather indicates blowing snow or any sort of precipitation (drizzle, rain or snow) these dates are classified as cyclonic conditions even when the current weather does not show cyclonic influence anymore.

Fog conditions do not necessarily mean more humid conditions close to the surface than the other noncyclonic conditions, for example, fog can form in very cold anticyclonic weather with low absolute, but high relative humidity. However, fog conditions prevail only for a small percentage of the study period and its small sample does not significantly influence the overall noncyclonic statistics. Table 1 presents the exact SYNOP code numbers defining the classification.

**TABLE 1:** Classification of the weather condition based on synoptic weather observations

	Cyclonic conditions	Noncyclonic conditions
Present weather (ww)	20–29\{28}: Precipitation at the station during the preceding hour	0–19: Cloud cover, haze and nonprecipitation events
	30–39: Blowing and drifting snow	28: Fog at the station during the preceding hour
	50–59: Drizzle	40–49: Fog and ice fog
	60–69: Rainfall	76–78: Diamond dust, snow grains and star like crystals during fog conditions
	70–79\{76,77,78}: Solid precipitation	
	80–89: Showers	
Past weather (WW)	3: Blowing snow	0–2: Cloud cover
	5: Drizzle	4: Fog
	6: Rain	
	7: Snow or mixed rain and snow	
	8: Showers	

### 2.5 Detection of temperature and humidity inversions

Specific humidity was calculated from the observed temperature, relative humidity and air pressure. The definition and terminology used to define temperature and humidity inversions generally follow that of Andreas *et al*. (2000), which was also used by Kilpeläinen *et al*. (2012), Nygård *et al*. (2013) and Vihma *et al*. (2011). However, different thresholds were defined. Two example temperature and humidity profiles are shown in Figure S10 together with the identified inversion layers. For the detection of inversion base and top we created a 5‐point moving average profile so that problems due to signal noise were avoided. This was done for both temperature and humidity profiles. The actual temperature and humidity values for the inversion base and top were then taken from the unsmoothed raw data profiles. The difference between the base height (*z*_*qb*_), where specific humidity starts vertically increasing, and the top height (*z*_*qt*_), where the specific humidity starts decreasing is the humidity inversion depth (∆*z*_*q*_), which makes ∆*z*_*q*_ = *z*_*qt*_ − *z*_*qb*_. The difference in specific humidity between the inversion top (*q*_*t*_) and base (*q*_*b*_) is the humidity inversion strength (∆*q*), meaning ∆*q* = *q*_*t*_ − *q*_*b*_. Although previous studies seldom consider a humidity strength threshold but only a positive vertical gradient, here, all humidity inversions deeper than 10 m and stronger than 0.1 g·kg^−1^ were detected, in order to avoid artificial inversions generated by sporadic inaccurate measurements related to, for example, a dry bias. Separated inversion layers are considered when there is a layer of negative vertical gradient in between inversion layers deeper than 100 m. Under cyclonic conditions, particularly in case of elevated inversions, the involved temperatures can be considerably higher than previously prevailing surface temperatures due to the advection of warm and moist air masses. Therefore, it is not necessarily the case that the temperature, and thus specific humidity, is lower at higher levels than at the surface, and in winter temperatures and specific humidity values similar to warmer seasons are found (e.g., Bagheri Dastgerdi *et al*., 2020). Consequently, we simply used the absolute values of specific humidity, a method widely used in earlier studies (e.g., Devasthale *et al*., 2011; Vihma *et al*., 2011; Kilpeläinen *et al*., 2012; Nygård *et al*., 2013).

Temperature inversions were detected analogously to humidity inversions. Hence, temperature inversion strength is ∆*T* = *T*_*t*_ − *T*_*b*_ and temperature inversion depth is ∆*z*_*T*_ = *z*_*Tt*_ − *z*_*Tb*_. But, only cases where the temperature inversion strength was larger than 1°C were of interest. Differences lower than the considered threshold may occur, but regarding their weakness they were ignored.

The upper limit of the lower troposphere is here considered to be at 500 hPa (~5,000 m). Since BSRN profiles comprise on average almost 100 vertical levels between the surface and 5,000 m, inversions not containing at least one level in between the inversion base and top were considered negligible.

Since the formation mechanisms are different for surface‐based and elevated inversions, three different inversion levels of occurrence were defined dependent on inversion base height: surface‐based if its base is located below 50 m; second‐level inversion if its base lies between 50 and 2,500 m; and third‐level inversions for all cases with base heights larger than 2,500 m. When more than one inversion of the same type exists in a profile, it is called multiple inversion.

## 3 RESULTS

**FIGURE 3:**
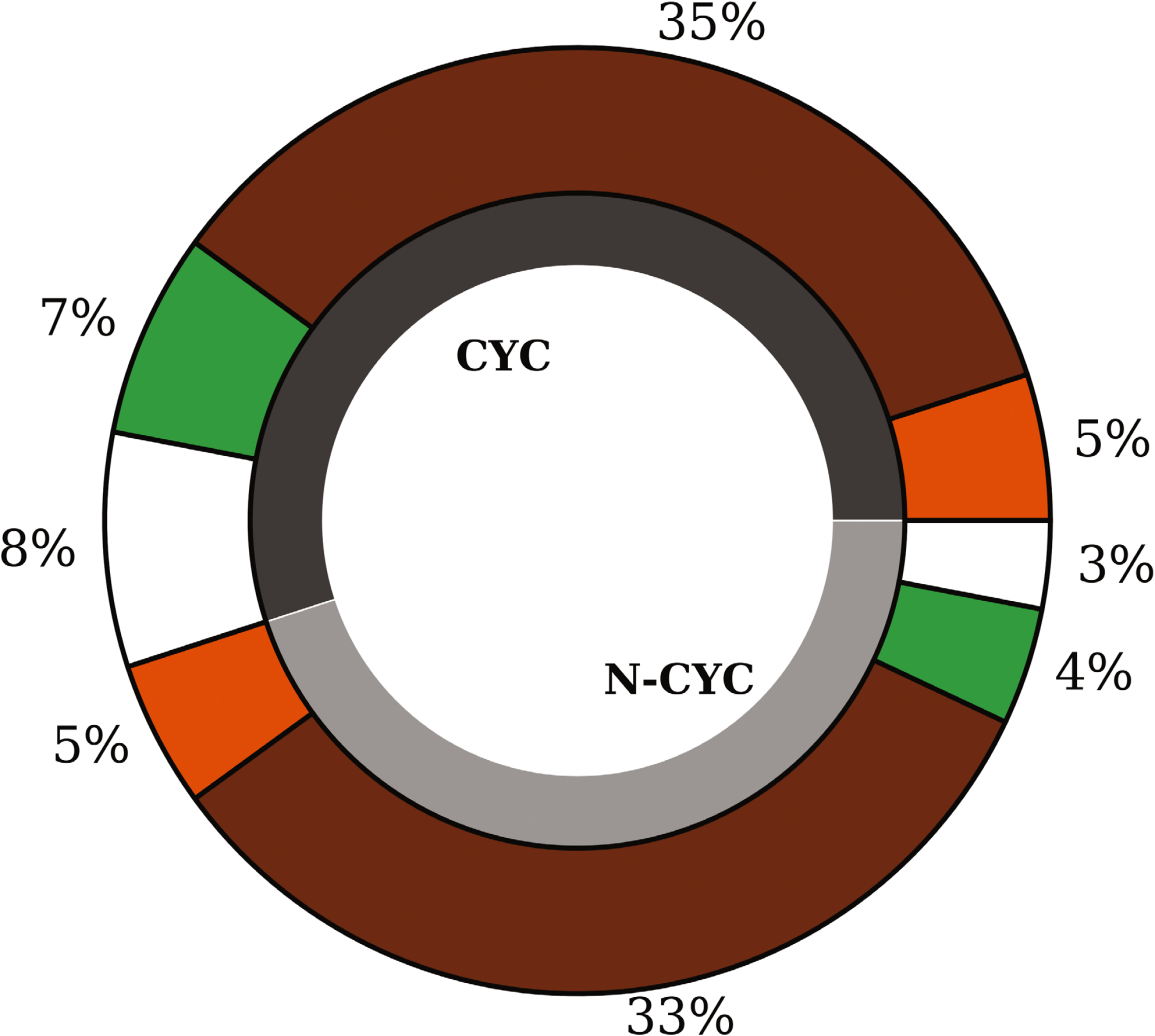
Total percentage of days with temperature (orange), humidity (green), simultaneous (brown) and without (white) inversions for cyclonic (CYC) and noncyclonic (N‐CYC) conditions [Colour figure can be viewed at wileyonlinelibrary.com]

For the time period 1993–2017, 8,489 radiosonde launches were analysed. Radiosondes were available for 93% of the times in a total of 9,131 days. Fifty‐five percent of these days were characterized by cyclonic, 45% by noncyclonic conditions (Figure 3). The most frequent cause of a radiosonde launch failure is high wind speeds, as proven by a success rate of 98% under noncyclonic versus 90% for cyclonic events.

### 3.1 General inversion occurrence

Note that in order to detect these temperature and humidity inversions, certain thresholds and definitions were applied (section 2.5), which shapes the presented results.

Following those definitions, Figure 3 shows that, overall, 78% of the profiles exhibit at least one temperature inversion (40% under cyclonic, 38% noncyclonic conditions). At least one humidity inversion was found in 79% of the profiles (42% cyclonic, 37% noncyclonic). Temperature and humidity inversions occurred simultaneously in about two thirds of the cases, in almost equal percentages for cyclonic and noncyclonic conditions. Multiple inversions in a profile are common in all seasons for both weather conditions.

**FIGURE 4:**
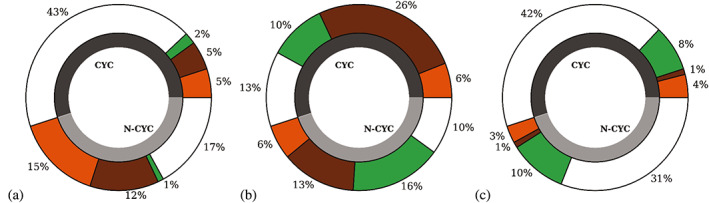
Percentage of days with temperature (orange), humidity (green), simultaneous (brown) and without (white) inversions at the three levels (first level: a; second level: b; third level: c) for cyclonic (CYC) and noncyclonic (N‐CYC) conditions; grey shaded area indicates the percentage of days under the prevailing weather condition [Colour figure can be viewed at wileyonlinelibrary.com]

In Figure 4 the overall percentages were split for the three different inversion levels. In total there are more temperature inversions at the second level (51%) than close to the surface (37%) and surface‐based inversions are found approximately three times as often under noncyclonic than under cyclonic conditions. Humidity inversions are generally more common at the second level than at the surface, with a similar relative frequency of occurrence for both weather conditions. At the surface level, temperature inversions are more frequent than humidity inversions for cyclonic and noncyclonic conditions, whereas at upper levels the contrary is found.

At the second level, while under noncyclonic conditions a large part of humidity inversions occur alone, for cyclonic conditions they are often associated with temperature inversions.

At the third level, inversions are generally rare and neither inversion type nor simultaneity depends on the weather conditions. For both cyclonic and noncyclonic conditions, they are always associated with an inversion underneath, most often with one at the second level.

Simultaneously occurring temperature and humidity inversions are mostly found at the surface level for noncyclonic conditions, whereas for cyclonic conditions they are more frequent at the second level.

### 3.2 Seasonality of inversion occurrence

In the following, the seasonality of the various features characterizing inversions will be investigated, separately for temperature and humidity inversions and for the different levels. Since we calculate monthly statistics rather than seasonal statistics, we do not define seasons here; however, note that winter in Antarctica usually comprises more than the 3 months of June, July and August. For coastal stations, which do not exhibit the coreless winter typical for inland locations (i.e., no distinct temperature minimum, but similar temperatures from late fall to early spring), a sharp definition of seasons does not seem to make sense since it would vary from year to year. For better flow of reading we preferred to use the word winter in the discussion rather than always specifying certain ranges of months. Generally, as we stated in section 2.1, cyclonic conditions are more frequent in spring and fall than in summer and winter. If we consider only days with inversions, this seasonality is still found, only slightly less pronounced.

**FIGURE 5:**
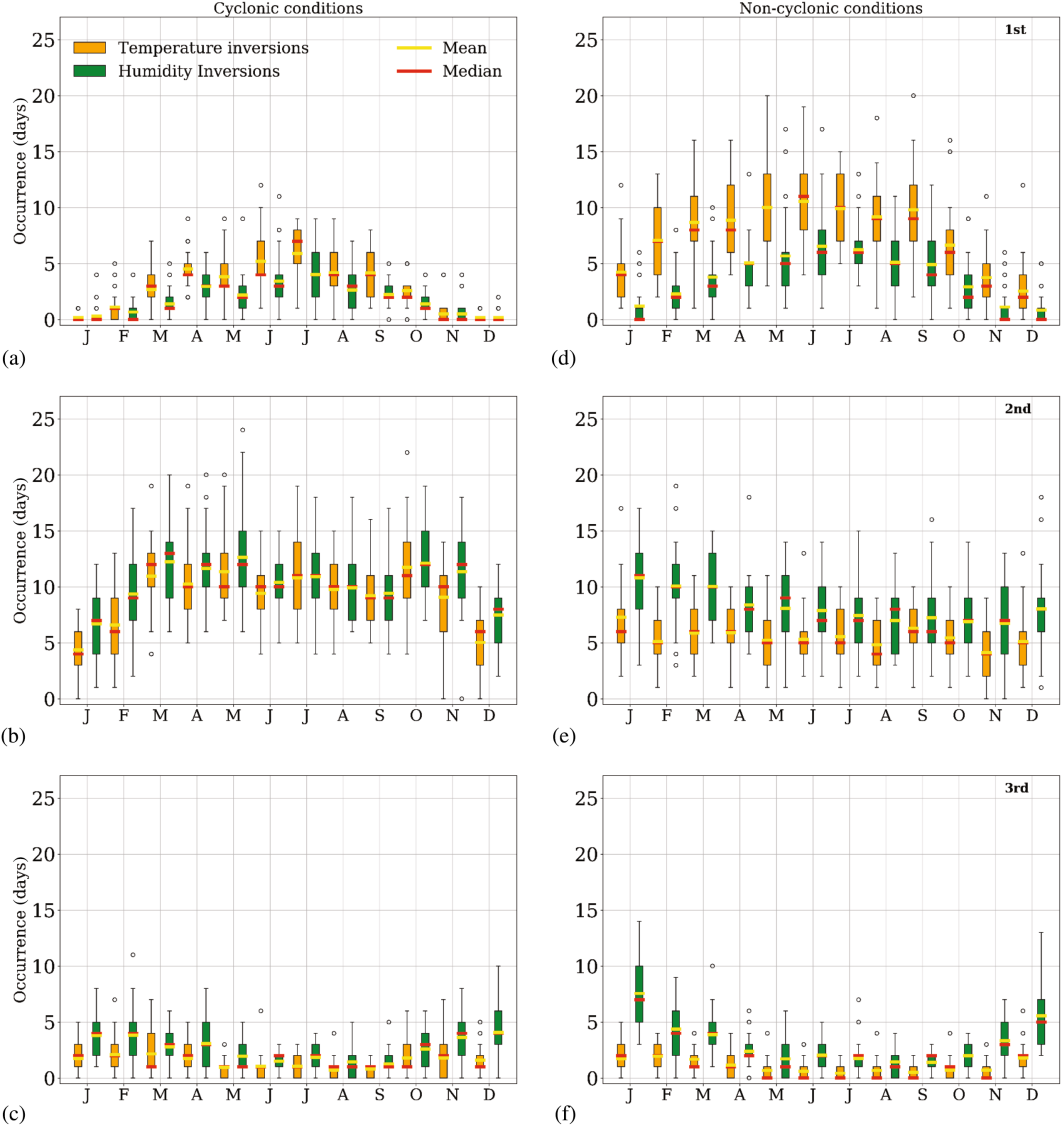
Monthly mean (yellow) and median (red) inversion occurrence (number of days with inversion) at the three different levels (first level: a, d; second level: b, e; third level: c, f) for cyclonic (a–c) and noncyclonic (d–f) conditions. The box contains all data between the 25th and 75th percentiles, separated by the median. As many of these variables are not normally distributed and likely to be affected by outliers, both mean and median values are displayed. Whiskers mark the limits of the lower and upper inner fence, defined as 1.5 IQR beyond the 25th and 75th percentiles, respectively. Outliers are defined as values outside the lower (upper) inner fence and marked individually [Colour figure can be viewed at wileyonlinelibrary.com]

In Figure 5, the monthly distribution of temperature and humidity inversion occurrence is shown for the three levels defined in section 2.5 under cyclonic and noncyclonic conditions. Figures S3 and S4 show a detailed view of the monthly distribution of days with temperature and humidity inversions. The box in Figure 5 contains all data between the 25th and 75th percentiles, separated by the median. As many of these variables are not normally distributed and likely to be affected by outliers, both mean and median values are displayed. Whiskers mark the limits of the lower and upper inner fence, defined as 1.5 times the interquartile range beyond the 25th and 75th percentiles, respectively. Outliers are defined as values outside the lower (upper) inner fence and marked individually.

At the surface level, generally temperature inversions are more frequent than humidity inversions. Both have a clear seasonal cycle with a minimum in summer and maximum in winter. This cycle is more pronounced for noncyclonic than for cyclonic conditions, the latter generally showing higher variability of occurrence. In all seasons, more inversions occur under noncyclonic (Figure 5d) than under cyclonic conditions (Figure 5a) at this level; in summer hardly any inversions are seen under noncyclonic conditions.

Contrary to the surface level, the second level exhibits more humidity inversions than temperature inversions and the inversion occurrence is higher for cyclonic (Figure 5b) than for noncyclonic conditions (Figure 5e). Under noncyclonic conditions, variability of occurrence is high, and no clear seasonal cycle is found here, whereas for cyclonic conditions maxima are seen in spring and fall, which are the seasons with the highest cyclonic activity.

At the third level, inversion occurrence is considerably lower than at the other two levels, with a mostly weak annual cycle that exhibits a minimum in winter. Only for humidity inversions under noncyclonic conditions (Figure 5f) a strong annual cycle can be seen that is contrary to the annual cycle at the surface, that is, a maximum of occurrence in summer and a minimum in winter.

### 3.3 Seasonality of temperature‐inversion features

**FIGURE 6:**
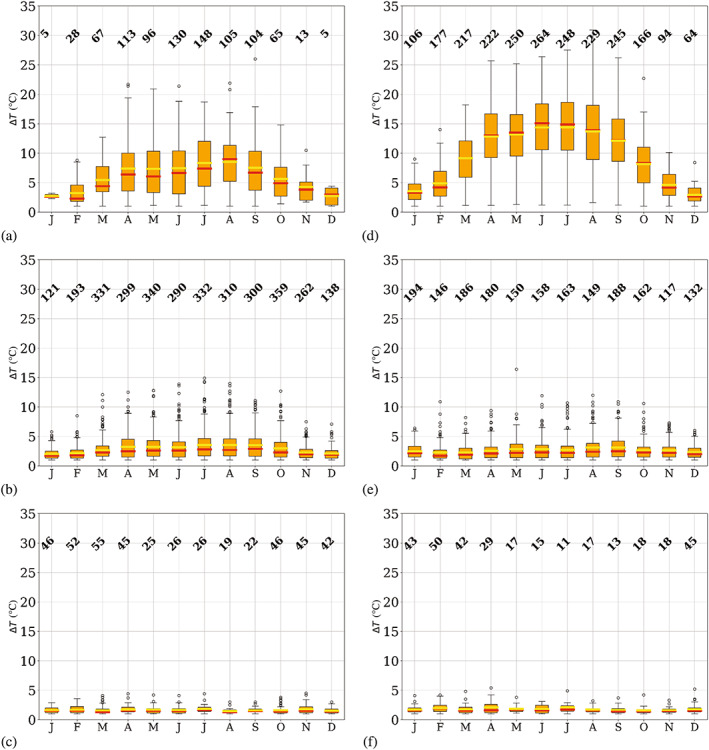
Monthly mean (yellow) and median (red) temperature inversion strength at the three different levels (first level: a, d; second level: b, e; third level: c, f) for cyclonic (a–c) and noncyclonic (d–f) conditions; numbers at the top of each subplot indicate the monthly sample size. Boxplots are described in Figure 5 [Colour figure can be viewed at wileyonlinelibrary.com]

**FIGURE 7:**
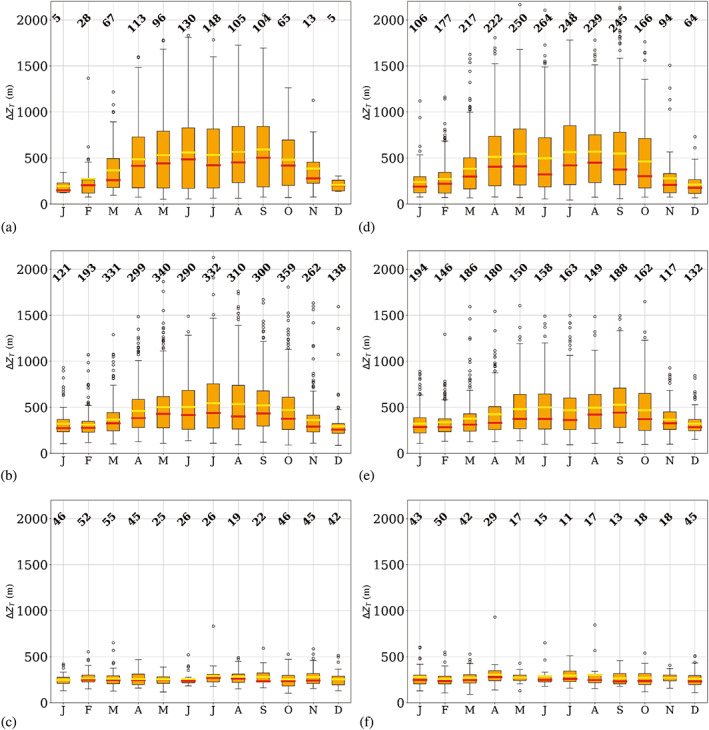
Monthly mean (yellow) and median (red) temperature inversion depth at the three different levels (first level: a, d; second level: b, e; third level: c, f) for cyclonic (a–c) and noncyclonic (d–f) conditions; numbers at the top of each subplot indicate the monthly sample size. Boxplots are described in Figure 5 [Colour figure can be viewed at wileyonlinelibrary.com]

**FIGURE 8:**
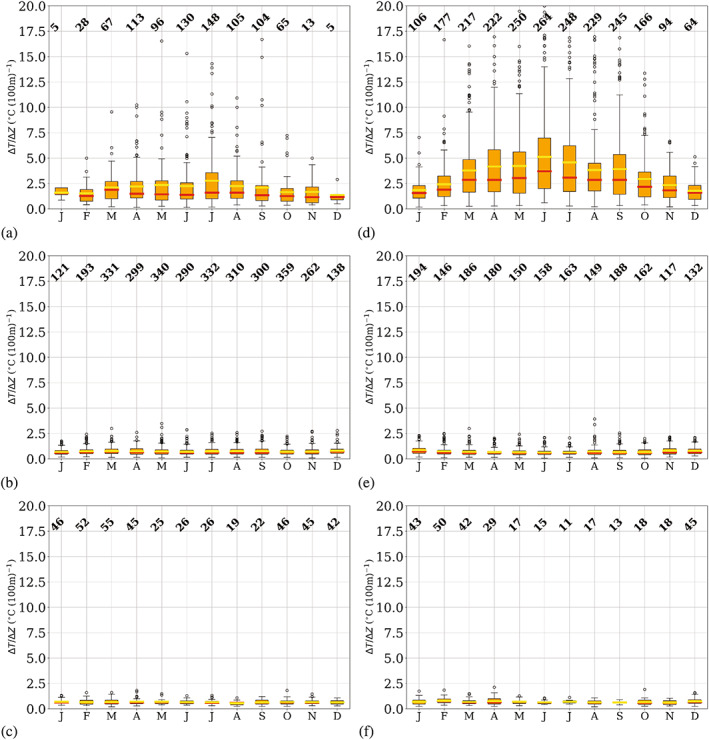
Monthly mean (yellow) and median (red) temperature inversion vertical gradient at the three different levels (first level: a, d; second level: b, e; third level: c, f) for cyclonic (a–c) and noncyclonic (d–f) conditions; numbers at the top of each subplot indicate the monthly sample size. Boxplots are described in Figure 5 [Colour figure can be viewed at wileyonlinelibrary.com]

The monthly distribution of temperature inversion occurrence is intrinsically related to the monthly temperature inversion features, that is, strength (∆*T*), depth (∆*Z*_*T*_) and vertical temperature gradient (∆*T*/∆*z*), which are shown in Figures 6, 7, 8, respectively.

In Figure 6, temperature inversion strength for the three levels and two weather conditions is displayed. Surface‐based inversions show a clear annual cycle with a winter maximum. The cycle is stronger and clearer for noncyclonic conditions (Figure 6d). Here in winter, values up to 25°C can be reached, whereas under cyclonic conditions (Figure 6a) the strength of inversions seldom exceeds 15°C. The differences in inversion strength between cyclonic and noncyclonic conditions are statistically significant at the 0.01 level between February and October (Table S1). The difference between mean and median values is smaller for noncyclonic conditions because generally higher values with less extreme outliers occur.

At the second level, inversions are considerably weaker than at the surface level, and only a very weak annual cycle shaped by outliers is found for cyclonic conditions (Figure 6b) with a winter maximum, whereas for noncyclonic conditions (Figure 6e) no annual cycle is seen. In contrast to the surface‐based inversions, cyclonic inversions are slightly stronger than noncyclonic ones, particularly in fall and winter. Only in summer, the noncyclonic inversions are slightly stronger than cyclonic inversions, whereas in winter the opposite is the case. Generally, strength differences between the two weather conditions are almost imperceptible and the differences are not statistically significant throughout most of the year (Table S1). The mean value is slightly higher than the median for both conditions in all seasons due to numerous outliers. The third level exhibits only very weak inversions, with no annual cycle, independent of the weather conditions.

Inversion depth is displayed in Figure 7. Surface‐based inversions show a relatively clear annual cycle with maxima in the polar night and no striking differences between the two weather conditions. During long, cold periods, noncyclonic conditions (Figure 7d) can lead to inversions deeper than 2,000 m. Those untypically thick temperature inversions are reflected in the large number of upper outliers, which leads to a difference between median and mean values of up to several hundred meters.

At the second level, inversions are generally less deep, thus the annual cycle is less pronounced. However, variability of inversion depth seems to be very high, which is demonstrated by the larger number of outliers for both weather conditions throughout the year.

Third‐level inversions are, with an approximate depth of 200 m, generally shallow and exhibit no annual cycle.

They are on average found at heights above 3,200 m and are independent of the weather conditions.

In Figure 8, the vertical gradient for temperature inversions at the corresponding level and weather conditions is shown. Largest gradients are found for surface‐based inversions under noncyclonic conditions (Figure 8b), where in winter values can reach up to 20°C (100 m)^−1^. Minimum values are found in summer for both weather conditions; the vertical gradients are one order of magnitude smaller than in winter. A general, slightly irregular annual cycle, however, is only found for noncyclonic conditions. A high number of outliers for almost all months for both weather conditions explains the relatively large differences between mean and median vertical temperature gradients, where the mean is always higher than the median.

At the second and the third level, vertical temperature gradients are generally low and almost constant throughout the year, with values of approximately 0.6°C (100 m)^−1^. Generally, values hardly vary more than 2°C (100 m)^−1^ throughout the year. Note that although inversions at the third level are considerably less deep than those at the second level, vertical gradients are similar at both levels because inversion strength at the third level is generally low, too.

### 3.4 Seasonality of humidity‐inversion features

**FIGURE 9:**
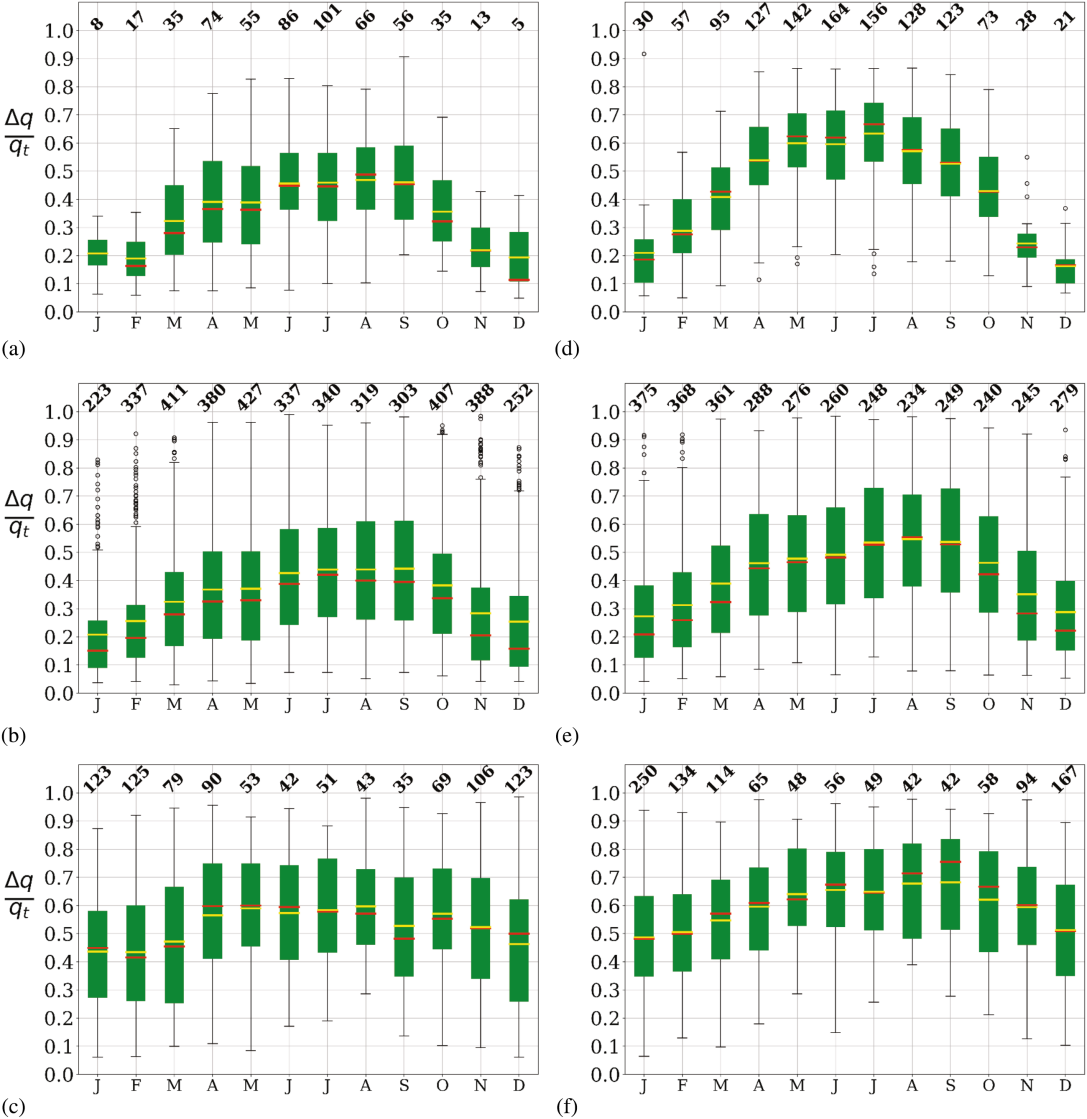
Monthly mean (yellow) and median (red) relative humidity inversion strength at the three different levels (first level: a, d; second level: b, e; third level: c, f) for cyclonic (a–c) and noncyclonic (d–f) conditions; numbers at the top of each subplot indicate the monthly sample size. Boxplots are described in Figure 5 [Colour figure can be viewed at wileyonlinelibrary.com]

**FIGURE 10:**
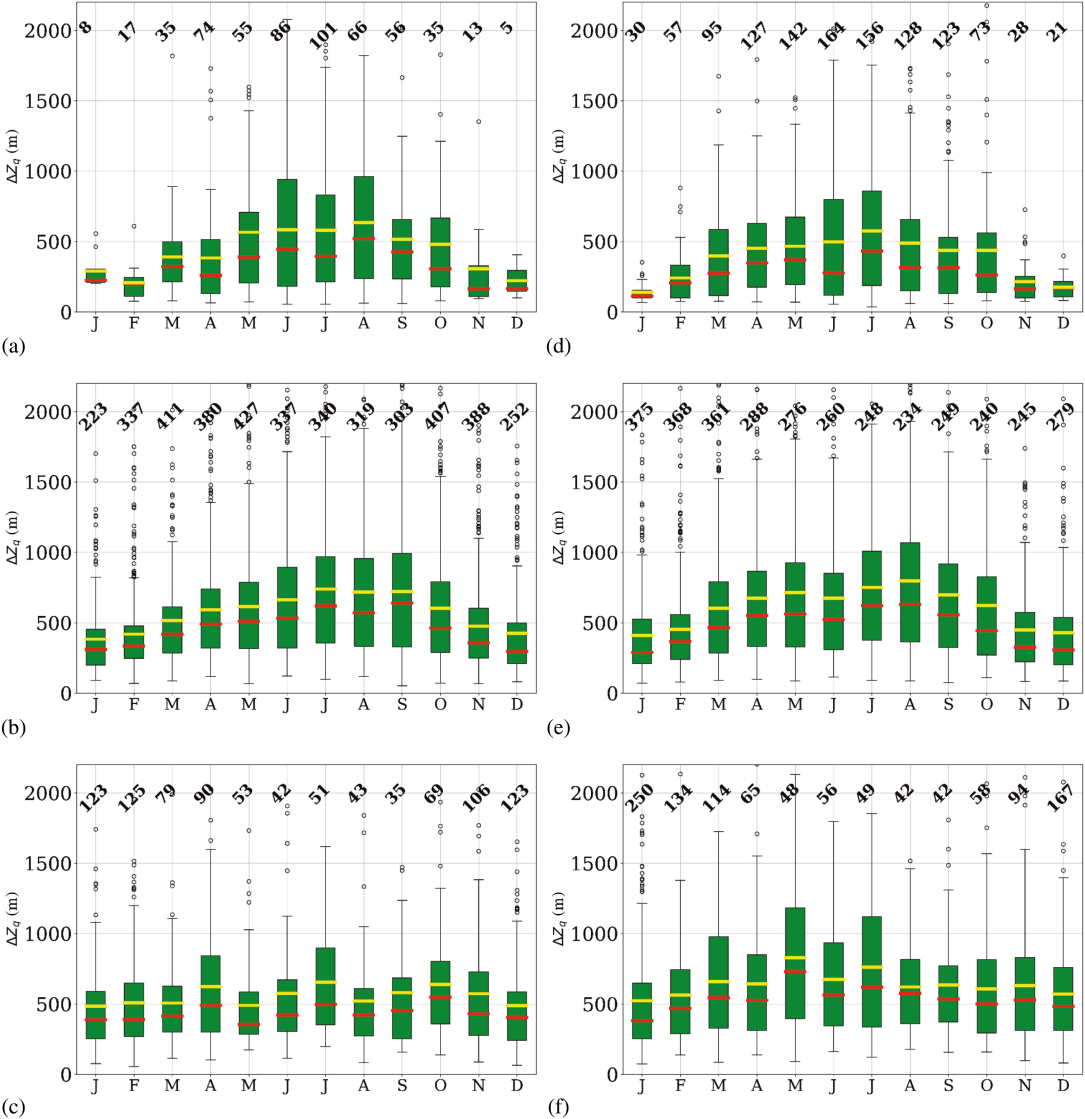
Monthly mean (yellow) and median (red) humidity inversion depth at the three different levels (first level: a, d; second level: b, e; third level: c, f) for cyclonic (a–c) and noncyclonic (d–f) conditions; numbers at the top of each subplot indicate the monthly sample size. Boxplots are described in Figure 5 [Colour figure can be viewed at wileyonlinelibrary.com]

**FIGURE 11:**
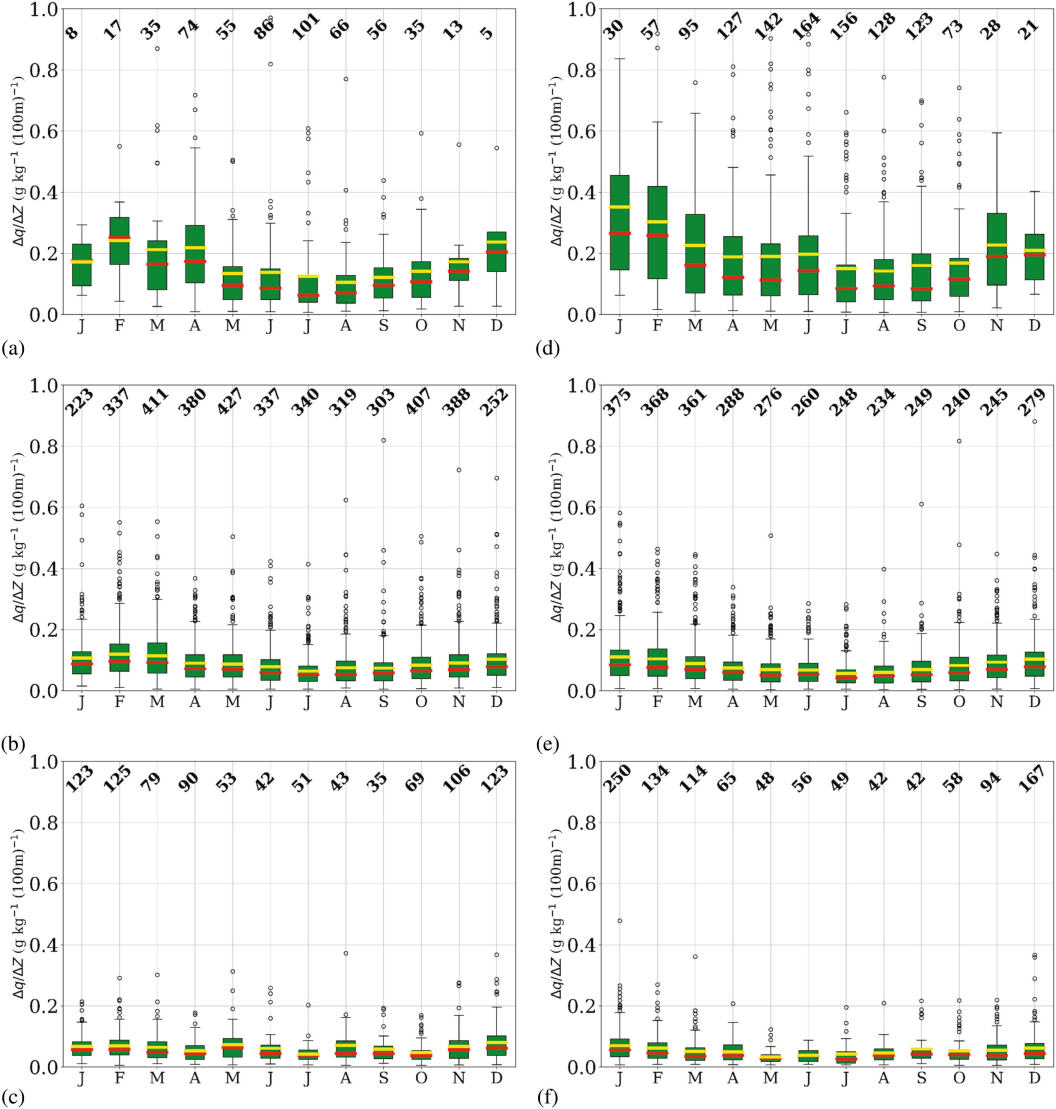
Monthly mean (yellow) and median (red) humidity inversion vertical gradients rate at the three different levels (first level: a, d; second level: b, e; third level: c, f) for cyclonic (a–c) and noncyclonic (d–f) conditions; numbers at the top of each subplot indicate the monthly sample size. Boxplots are described in Figure 5 [Colour figure can be viewed at wileyonlinelibrary.com]

Since humidity is strongly related to temperature via the Clausius–Clapeyron equation, we introduced a relative humidity inversion strength normalized by the specific humidity at the top of the inversion. We included Figure S5 that shows the absolute humidity inversion strength (∆*q*). Figures 9, 10, 11 present the monthly relative humidity inversion strength (∆*q*/*q*_*t*_), depth (∆*z*_*q*_) and vertical gradient (∆*q*/∆*Z*), respectively.

Relative inversion strength shows a pronounced seasonal cycle at all three vertical levels and during both cyclonic and noncyclonic conditions, with overall higher values during wintertime (Figure 9). Since specific humidity undergoes equally strong seasonal changes, the seasonal cycle of the absolute inversion strength is less pronounced and more variable than the normalized strength (Figure S5). The same effect can also be seen in the change of inversion strength with height. While the absolute strength decreases with height, the magnitude of the relative strength is relatively similar at all levels because of a simultaneous decrease of absolute humidity with height. The seasonal cycle with stronger inversions during wintertime does, however, become less pronounced with height, resulting only in a weak seasonal cycle at the third level (Figure 9). At the same time, the variability in strength increases with height. Numerous values above the 75th percentile demonstrate that humidity inversions can actually be stronger at the second and third level than at the surface, particularly in summer.

For noncyclonic conditions, the time of the peak in inversion strength shifts slightly with height from winter at the first level towards early spring at the second and third level. Since absolute inversion strength shows a different seasonal behaviour at the second level, with a peak during fall (Figure S5), this suggests that the spring peak in relative strength occurs in connection with relatively low values in specific humidity. This might be related to the maximum in sea ice extent, which usually occurs in September.

Similar to temperature inversions, surface‐level noncyclonic inversions are somewhat stronger than cyclonic inversions, although the difference is less pronounced than for temperature inversions. The same is also true for the second‐level humidity inversions. Since specific humidity is also higher during cyclonic conditions, the absolute inversion strength does, however, not differ significantly between cyclonic and noncyclonic conditions (Figure S5 and Table S2).

Humidity inversion depth (Figure 10) shows a clear annual cycle with winter maxima and summer minima at the first and second level for both weather conditions, which is similar to the annual cycle of temperature inversions. However, in strong contrast to temperature inversions, humidity inversions at the third level have depths similar to the two other levels and, at both upper levels, humidity inversions are deeper than the corresponding temperature inversions, independent of the weather condition. No clear annual cycle is found at the third level.

The vertical humidity gradient (Figure 11), similar to temperature, shows high values at the surface level, whereas elevated humidity inversions exhibit fairly low gradients. The annual cycle is opposite to the cycle for temperature inversions, with maxima in summer and minima in winter for both weather conditions. Higher values occur for noncyclonic conditions (Figure 11d), particularly in summer, where also variability is highest, so that these differences are not statistically significant (Table S2).

At the second level, generally low values with a weak annual cycle are found, and at the third level the annual cycle cannot be detected anymore. The large inversion depth combined with moderate to low strength leads to the fairly low vertical gradient, with values mostly below 0.1 g·kg^−1^ (100 m)^−1^.

Differently from temperature inversions, humidity inversion strength, depth and vertical gradient have rather different annual cycles. A strong humidity inversion is not necessarily also a deep inversion. For example, the wintertime maximum in inversion depth for surface‐based inversions leads to a minimum in the vertical gradient in the absence of a pronounced seasonal cycle in inversion strength. Although absolute values of specific humidity are comparatively small in winter because of the low saturation vapour pressure, these results clearly show that, as stated in section 2.5, the temperature dependence of specific humidity is not the dominant factor in the seasonality of humidity inversion properties.

### 3.5 Monthly composite profiles

**FIGURE 12:**
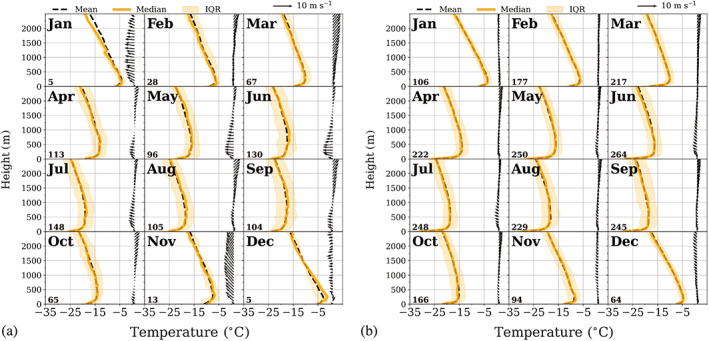
Vertical profiles of the monthly mean (solid) and median (dashed) temperature that detected surface‐based temperature inversions. The interquartile range (IQR) is shaded. The associated mean wind vector (arrows) is in intervals of 100 m for cyclonic (a) and noncyclonic (b) conditions. The numbers of cases are given in the lower left corner for each month [Colour figure can be viewed at wileyonlinelibrary.com]

**FIGURE 13:**
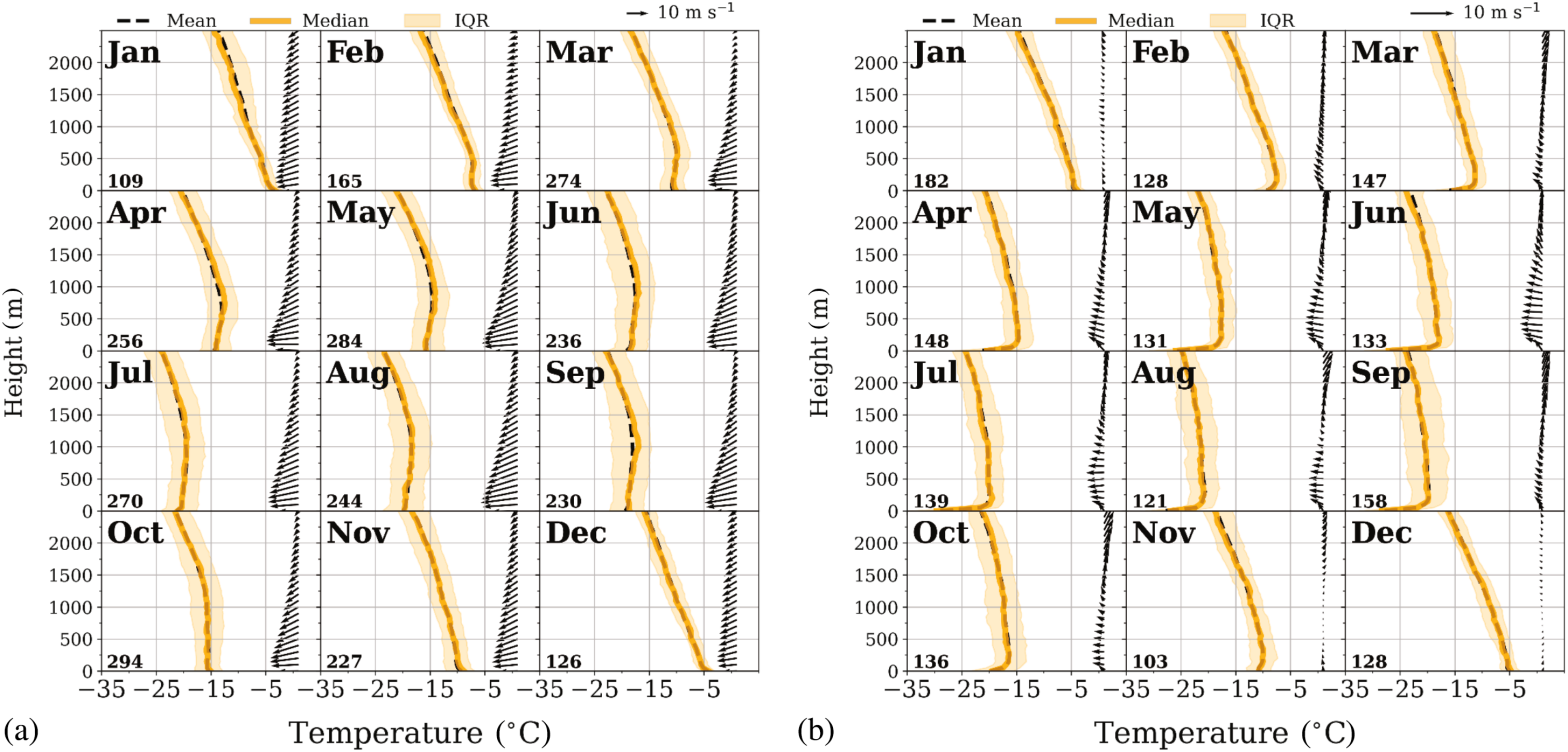
Vertical profiles of the monthly mean (solid) and median (dashed) temperature that detected temperature inversions at the second level. The interquartile range (IQR) is shaded. The associated mean wind vector (arrows) is in intervals of 100 m for cyclonic (a) and noncyclonic (b) conditions. The length of the wind vectors differs between (a) and (b). The respective length scales are shown in the top right corner of each panel. The numbers of cases are given in the lower left corner for each month [Colour figure can be viewed at wileyonlinelibrary.com]

Mean monthly vertical profiles of temperature and humidity were analysed up to 2,500 m, thus including surface‐ based inversions and inversions at the second level. In order to calculate the mean vertical profiles, all atmospheric profiles containing at least one inversion at the corresponding level were isolated from the main data set and re‐ sampled in intervals of 50 m. Missing values were calculated by linear interpolation. These profiles were divided into 12 monthly subsets to produce monthly composites for both weather conditions. These composites together with the corresponding vertical wind profiles are shown in Figures 12 and 13 for temperature and 14 and 15 for humidity inversions. The shaded areas correspond to the range between the 25th and 75th percentile. The mean monthly wind vector is displayed in intervals of 100 m. We restrict the height to 2,500 m since, at the third level, inversions are so weak that they are not visible in mean (median) profiles.

The composite temperature profiles for days with surface inversions (Figure 12) and for days with inversions at the second level (Figure 13) for both cyclonic (a) and noncyclonic (b) conditions are displayed together with the mean monthly wind vector. Note that those divisions are not exclusive, that is, days with surface‐based inversions can have inversions at the second level, too, and vice versa. The numbers of cases are given in the lower left corner for each month.

For surface‐based inversions our earlier results are confirmed by the monthly profiles, the inversions are generally seen in the monthly mean, they are stronger in winter than in summer and also stronger for noncyclonic than for cyclonic conditions. Median and mean values are almost identical, except for cyclonic conditions in summer, where inversion depth and strength are generally smallest so that the influence of outliers on the mean is largest.

Winds are mostly weak and come from an easterly (southerly) direction under cyclonic (noncyclonic) influence. The cases with inversions under cyclonic conditions are usually at the beginning or, more often, at the end of a cyclonic weather situation, following our definition (section 2.5), where the weather between the actual SYNOP observation and the preceding observation is taken into account, too. Whereas a few precipitation events per year occur without strong winds, wind speeds usually increase considerably under the influence of a passing cyclone and surface‐based inversions are destroyed.

Inversions at the second level, however, are not always detectable in composites because they are either too weak or too variable in height and depth, particularly under noncyclonic conditions, where also only weak winds are observed. Strikingly different is the picture for elevated inversions under cyclonic conditions (Figure 13a). Except for the summer months, those inversions are clearly visible in monthly profiles. Wind speeds are considerably higher than in the three other cases. The profiles show strong easterly winds close to the surface, with a Low‐Level Jet (LLJ) below 500 m very common throughout the year, except for the summer months November–January. Wind speeds here are higher at the inversion base than at the top, with typical differences of 3 m·s^−1^, indicating the proximity of the LLJ to the inversion base. Moreover, this is the only level that fulfils the LLJ definition of Blackadar (1957). Wind direction changes from east to northeast, thus counter‐clockwise with height, associated with warm air advection. We calculated the percentage of inversions with counter‐clockwise rotation of the wind direction from the base to the top of the inversion for the different levels and weather conditions. Corresponding to the results described above, only for the second inversion level a noteworthy difference was found between cyclonic and noncyclonic conditions (66 and 42%, respectively). Similarly, understanding moisture transport as the simple product of specific humidity and wind speed (e.g., Nygård *et al*., 2013; Gorodetskaya *et al*., 2020), a significant upward increase of moisture transport within the inversion was only found for elevated inversions.

**FIGURE 14:**
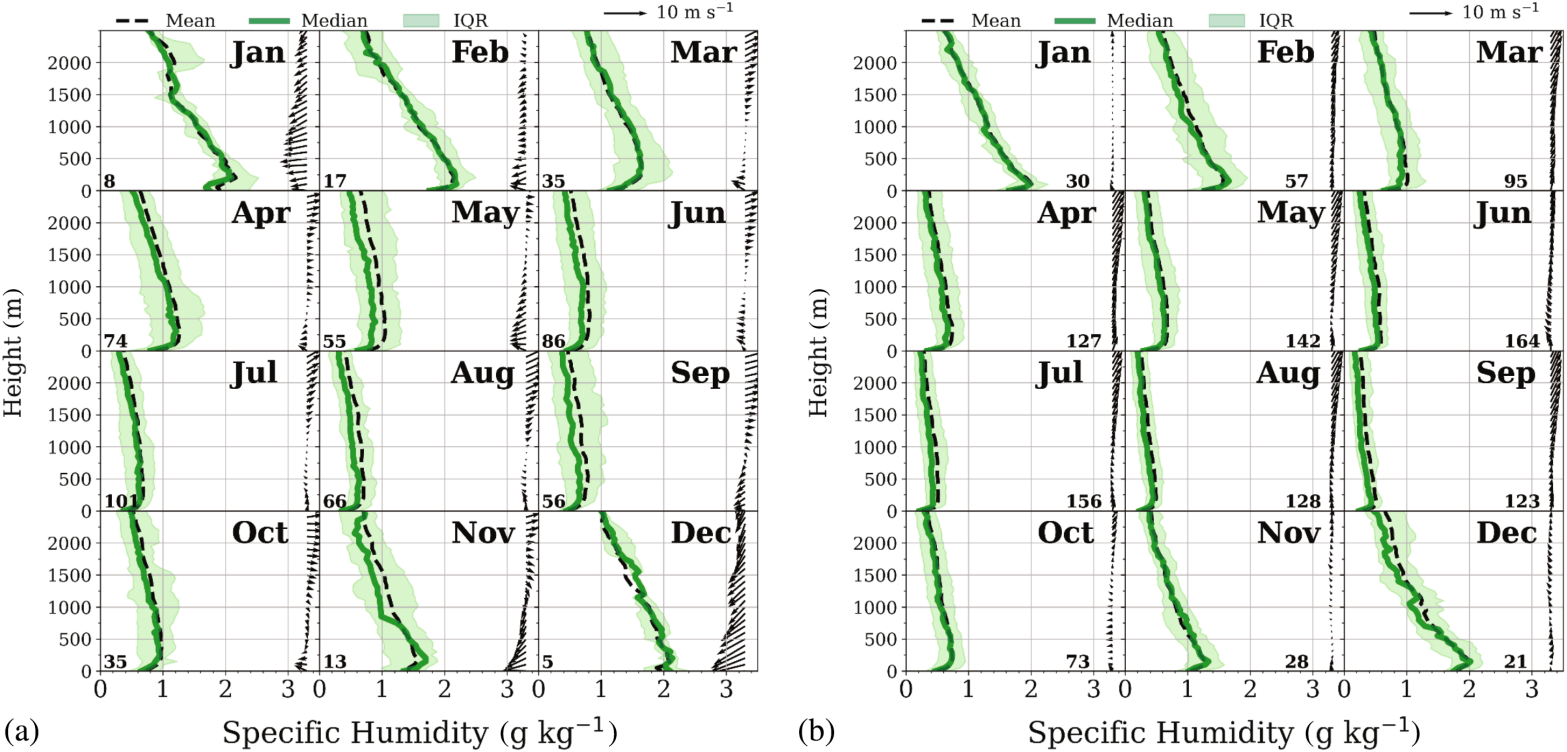
Vertical profiles of the monthly mean (solid) and median (dashed) specific humidity that detected at surface‐based humidity inversions. The interquartile range (IQR) is shaded. The associated mean wind vector (arrows) is in intervals of 100 m for cyclonic (a) and noncyclonic (b) conditions. The numbers of cases are given in the lower left corner for each month [Colour figure can be viewed at wileyonlinelibrary.com]

**FIGURE 15:**
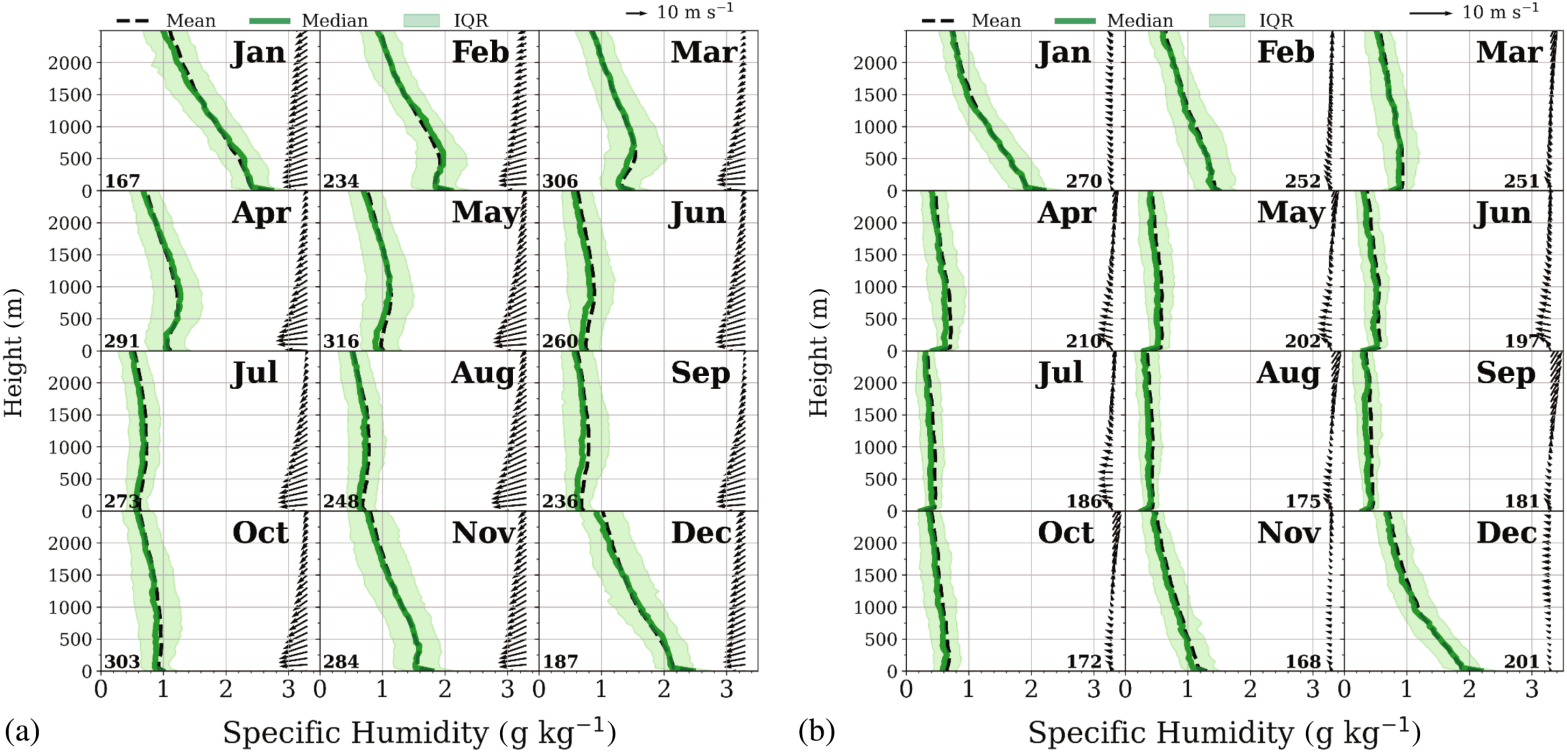
Vertical profiles of the monthly mean (solid) and median (dashed) specific humidity that detected humidity inversions at the second level. The interquartile range (IQR) is shaded. The associated mean wind vector (arrows) is in intervals of 100 m for cyclonic (a) and noncyclonic (b) conditions. The length of the wind vectors differs between (a) and (b). The respective length scales are shown in the top right corner of each panel. The numbers of cases are given in the lower left corner for each month [Colour figure can be viewed at wileyonlinelibrary.com]

The corresponding monthly composite humidity profiles (Figure 14—surface‐based inversions and Figure 15—inversions at the second level) are basically similar to the temperature profiles, which makes sense since temperature and humidity inversions at the second level are in the majority of cases simultaneous (Figure 5). Again, only for the second level under cyclonic conditions the wind plays an important role and a LLJ is observed. Generally, the humidity profiles show higher variability than the temperature profiles, particularly the surface‐based inversions exhibit less smooth profiles than the corresponding temperature profiles due to higher variability in elevation, depth and strength, which also leads to larger differences between median and mean values. Additionally, note the partly low number of cases per month.

**FIGURE 16:**
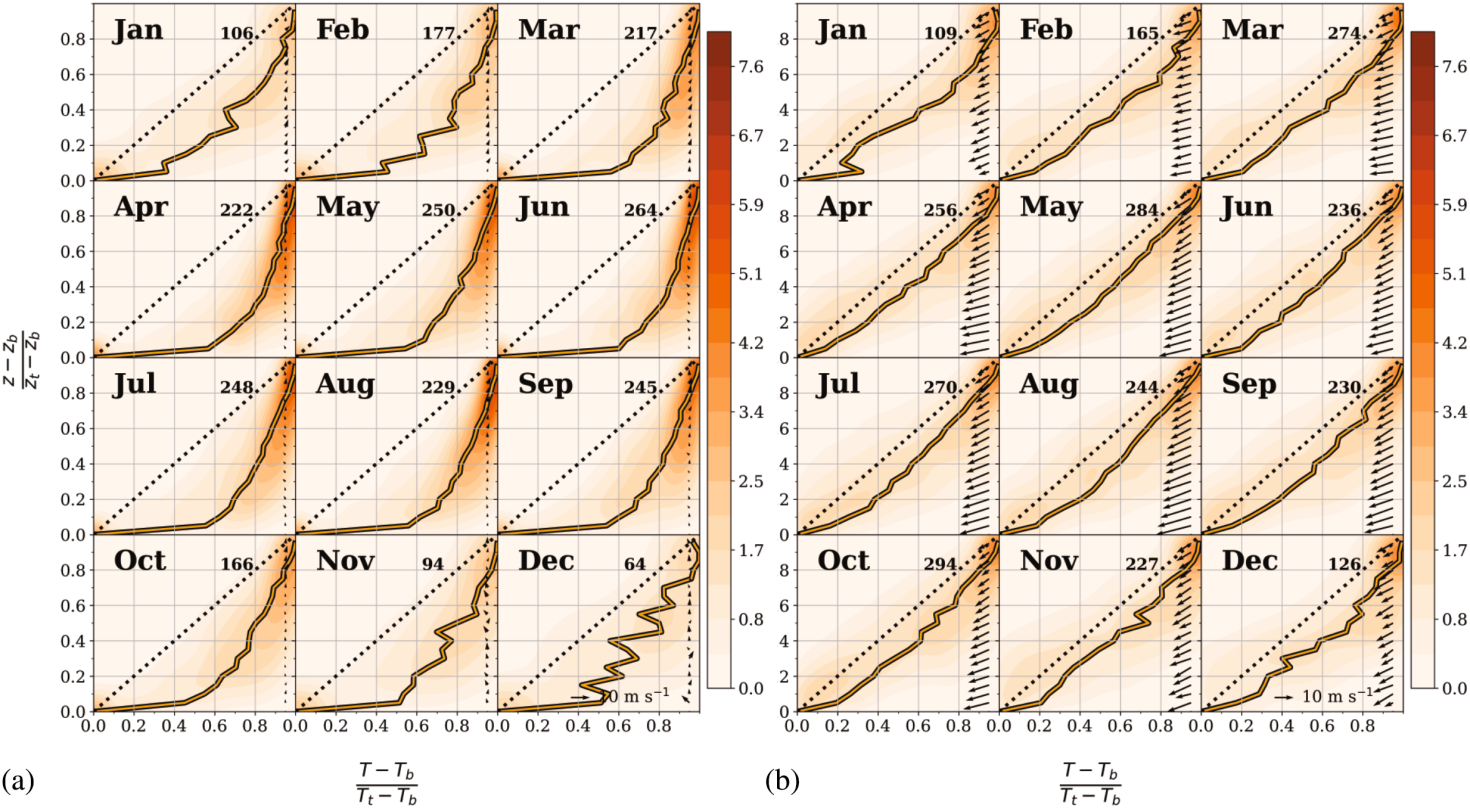
Monthly two‐dimensional probability density function (PDF) of temperature and height for surface‐based inversions under noncyclonic conditions (a) and for the second inversion level under cyclonic conditions (b). Temperature and height within the inversion layer were normalized by inversion strength and depth. Additionally, the mean vertical temperature profiles are shown. The normalized profile and the wind vector are vertically averaged in 0.05 bins. The 1:1 line represents a constant temperature rate increase with height within the inversion layer [Colour figure can be viewed at wileyonlinelibrary.com]

Due to varying inversion strength and depth, and, particularly for the elevated inversions, varying heights, the composite profiles tend to yield a blurred picture. Thus, additional profiles of the individual inversion layers are analysed to gain a better picture of the vertical structure within the inversions. The mean temperature profiles are shown for surface‐based inversions under noncyclonic conditions and for second‐level inversions under cyclonic conditions in Figure 16. In addition, all temperature and humidity profiles for both surface‐based and second‐level inversions and for cyclonic and noncyclonic conditions are included in Figures S6–S9. If multiple inversions occur at the second level, only the strongest inversion was selected. In the profiles, the height is normalized by the inversion depth and temperature and humidity are normalized by the respective inversion strength so that deep and shallow inversions can be averaged without producing any undesirable smoothing effects. To provide further information about the variability in the inversion structure, the figures also include the respective two‐dimensional probability density functions (PDF). The mean profiles and additional wind vectors are only shown for months with more than 30 inversions. No profiles are thus included for surface‐based inversions during cyclonic conditions in summer, which are overall relatively weak and shallow, and the wind speed and direction are highly variable.

For surface‐based inversions under noncyclonic conditions (Figure 16a), the mean profile shows that up to approximately 70% of the temperature increase with height occurs already in the lowest 20% of the inversion layer due to strong radiative cooling close to the surface under quiet wind conditions. The temperature inversions are thus characterized by a two‐layer structure with very strong stratification close to the surface and a weaker temperature gradient above. The high probability density surrounding the mean profile in the upper part of the inversion confirms that most of the individual profiles reach a temperature close to the temperature at the inversion top already near the middle of the inversion layer and thus show a vertical structure similar to the mean profiles.

At the second level under cyclonic conditions (Figure 16b) wind speeds are distinctly higher than for noncyclonic conditions and the direction changes with height from east‐northeast to northeast. The maximum wind speed within the inversion reaches 20 m·s^−1^. However, the absolute maximum of wind speed for the whole profile is not found within the inversion, but underneath. The LLJ is thus usually seen below the inversion (see also Figure 13a). The vertical structure of the inversion also differs from that of noncyclonic surface‐based inversions. While the temperature gradient in the mean profile is also slightly larger in the lowest 10–20% of the layer than further above, the profile is overall closer to the dotted 1:1 line, thus indicating a near‐constant temperature increase with height. The area of high probability density is similarly largely below the 1:1 line, but it also extends to above the line, which means that the gradient is stronger in the upper part of some of the inversions than in the lower part.

## 4 DISCUSSION AND CONCLUSION

For the first time, a 25‐year inversion climatology for Neumayer Station, Antarctica, was presented that takes into account different levels of occurrence and different weather situations. In spite of a relatively simple, dual weather classification clear differences were found for inversion features depending on inversion level and synoptic conditions. Also, formation mechanism depended on weather situation and level of occurrence.

Surface‐based and elevated inversions show distinctly different features that are related to the formation mechanisms of the inversions, which, in turn, clearly depend on the synoptic situation. This is the case for both temperature and humidity inversions. The relationship between the inversions at the different levels as well as between temperature and humidity inversions are rather complex.

Generally, surface‐based inversions develop under quiet conditions with low wind speeds, thus little or no turbulent mixing, due to the negative energy balance of the surface. Particularly in winter, when incoming short‐wave radiation is low or nonexistent in the polar night, strong inversions are found, corresponding to the winter maxima in general inversion occurrence as well as temperature inversion strength, depth and vertical gradients. Simultaneously occurring surface‐based temperature and humidity inversions are partly coupled via the Clausius–Clapeyron equation (Curry, 1983). However, since the atmosphere is usually not saturated, this is only one general factor. The amount of moisture in the SBL depends on weather history, because sublimation from the cold snow surface is small compared to horizontal advection of moisture. Under noncyclonic conditions, remainders of moisture advected previously by a passing cyclone can either increase long‐wave downward radiation, or, under very cold conditions, fall out as diamond dust. Surface‐based humidity inversions are additionally influenced by hoar frost formation, which reduces humidity close to the surface.

Elevated inversions, particularly at the second level and under cyclonic conditions, exhibit high wind speeds, often a LLJ from easterly directions. They are usually associated with the passing of cyclones with the corresponding frontal systems that cause advection of heat and moisture from lower latitudes. Warm air advection above the original cold air at lower levels can thus lead to strong temperature inversions, and the moisture advection causes even stronger and deeper humidity inversions at the second level. This is supported by the counter‐clockwise change of wind direction with height (Figures 13a and 15a).

The relationship between cyclonic activity and elevated inversion occurrence and features can also be seen in the spring and fall maxima of temperature and humidity inversion occurrence under cyclonic conditions at the second level. Here humidity inversion strength exhibits a maximum in spring, which is most likely also related to the spring maximum in baroclinicity (Van Loon, 1967). In contrast, humidity inversion depth shows a winter maximum also at the second level. The reason for the latter is not clear from our investigation, more detailed case studies would be necessary here.

Note that nocturnal inversions in summer could not be detected in the radiosonde launches carried out on a routine basis around noon at Neumayer, but are often observed in the morning after clear nights with low wind speed, clearly detectable by the strong mirages that they cause. These inversions are usually shallow, which is proved by the fact that they are often only visible from the ground and cannot be seen anymore from the station roof 17.5 m above the surface. Since they are also short‐lived they are not captured in our study, but we just like to mention them for the sake of completeness.

A comparison with earlier studies is only of limited feasibility since, as mentioned before, our study is the first to investigate a long time series of inversions with differentiation between different levels of occurrence and weather situations. Vignon *et al*. (2019) gave only rather general results for inversions on ice shelves, they focused on near‐surface winds and model evaluation. The main result for the two ice shelf stations, Neumayer and Halley, is that they are not influenced by katabatic winds, but Vignon *et al*. (2019) do not show any results that could be compared to our study.

Nygård *et al*. (2013) also included Neumayer in their study of humidity inversions at 11 Antarctic coastal stations. They had a different attitude, however, for example, they looked at the number of inversions per ascent rather than studying inversions at a certain level. However, both studies agree, that typically multiple inversions are observed. Nygård *et al*. (2013) also found that approximately half of the humidity inversions occurred simultaneously with temperature inversions, especially at the surface. Our study yielded higher percentages of simultaneous inversions at the first and second level, whereas, at the third level, humidity inversions associated with temperature inversions were rare. Humidity inversions were found to be most frequent in winter and spring, which corresponds to our results for surface‐based inversions under noncyclonic conditions. For elevated inversions and also for all inversions under cyclonic influence our results are different from those of Nygård *et al*. (2013). Note that also the chosen thresholds for identifying inversions differed from the ones used in this study. Our choice of thresholds was motivated by the interest in the influence of inversions on ice core interpretation. The third level is actually of minor practical importance here.

The use of BSRN (Driemel *et al*., 2018) versus IGRA (Integrated Global Radiosonde Archive) data (Durre *et al*., 2006) inhibits the direct comparison with the Nygård *et al*. (2013) study and also influences the results to a certain extent. As stated in section 2.1, IGRA data provide mostly mandatory pressure levels and thus have lower vertical resolution than the BSRN data. Given differences in the quality control, occasionally certain pressure levels in a profile were not available in one data set but in the other. For the same reason, Vignon *et al*. (2019) did not use IGRA data either, but requested the data from national agencies.

The division into cyclonic and noncyclonic conditions is a rather simple one and has its shortcomings, namely that in the transition times when a cyclone was just approaching or moving away, the definition is ambiguous. On the other hand, under noncyclonic conditions, particularly for humidity inversions the moisture remaining from the cyclone that had just passed plays a role. We chose the 25‐year period, although the change of sensors during this period might have led to inconsistencies, but this disadvantage is small compared to the advantage of using a considerably longer time series than previous studies.

The investigation of moisture transport was done in a rather simple way (described in section 3.5), which is nevertheless meaningful and has been used similarly by other authors (e.g., Dufour *et al*., 2019; Gorodetskaya *et al*., 2020). It is beyond the scope of our study to do a detailed calculation of advection terms using atmospheric model output.

Our results can be applied to most areas that have similar conditions as Neumayer Station, that is, ice shelf regions that are not strongly influenced by katabatic winds. 75% of the Antarctic coastline are fringed by ice shelves. They have a total area of approximately 1.5 × 10^6^ km^2^, of which ~1 × 10^6^ km^2^ correspond to the two large ice shelves, Filchner‐Ronne and Ross Ice Shelf, which are climatically different from the others since they are situated in deep embayments at a higher latitude than the rest of the Antarctic coast. The same is valid to a certain degree for Amery Ice Shelf. Most other ice shelves have a relatively small north–south extent and their weather and climate conditions are similar to those at Neumayer, that is, strongly influenced by the circumpolar trough. We expect similar inversion features here. This would involve, for example, stations like Maudheim, SANAE (I‐III), Halley, and large parts of the Antarctic coast that are not inhabited. Stations like Dumont D'Urville and Mawson, which endure strong katabatic winds, are not comparable.

In the interior of the continent, conditions are not directly comparable either, since they are less influenced by the synoptic activity in the circumpolar trough. However, whereas cyclones hardly ever penetrate into the interior, amplified Rossby waves can lead to strong heat and moisture advection from lower latitudes to the Antarctic plateau, which usually destroys the prevailing surface inversion, but can also lead to the formation of elevated inversions.

The 25‐year inversion climatology presented here is supposed to serve as a first step towards a better understanding of Antarctic inversion features, which will help to improve the current paleoclimatic interpretation of ice cores (e.g., Jouzel and Merlivat, 1984). We used monthly mean (median) values to do statistical calculations of inversion occurrence and features. For a more detailed analysis, higher temporal resolution combined with surface energy balance studies as well as a more complex weather situation classification would be recommended. Additionally, the use of model data to explicitly calculate moisture and heat advection would help to get a deeper insight into formation mechanisms, particularly for elevated inversions.

## AUTHOR CONTRIBUTIONS

Tiago Silva and Elisabeth Schlosser structured the study. Tiago Silva did the data analysis. Tiago Silva, Elisabeth Schlosser and Manuela Lehner interpreted the results. Tiago Silva, Elisabeth Schlosser and Manuela Lehner wrote the manuscript.

## CONFLICT OF INTEREST

The authors declare no potential conflict of interest.

## Supporting information


**Appendix S1** Supporting Information.

## Data Availability

All data sets of upper‐air soundings and synoptic weather observations covering the entire study period at Neumayer Station are publicly available either at PANGEA website (https://www.pangaea.de/) or at Baseline Surface Radiation Network (Driemel *et al*., 2018) website (https://bsrn.awi.de/).
