# Supplementary material for: A 25‐year climatology of low‐tropospheric temperature and humidity inversions for contrasting synoptic regimes at Neumayer Station, Antarctica
Source: Int J Climatol. 2022 Jul 10;43(1):456–79. doi: 10.1002/joc.7780 (PMC10084401; doi:10.1002/joc.7780)
Supplement: Supplementary file 1 — Appendix S1 Supporting Information. [file JOC-43-456-s001.pdf]

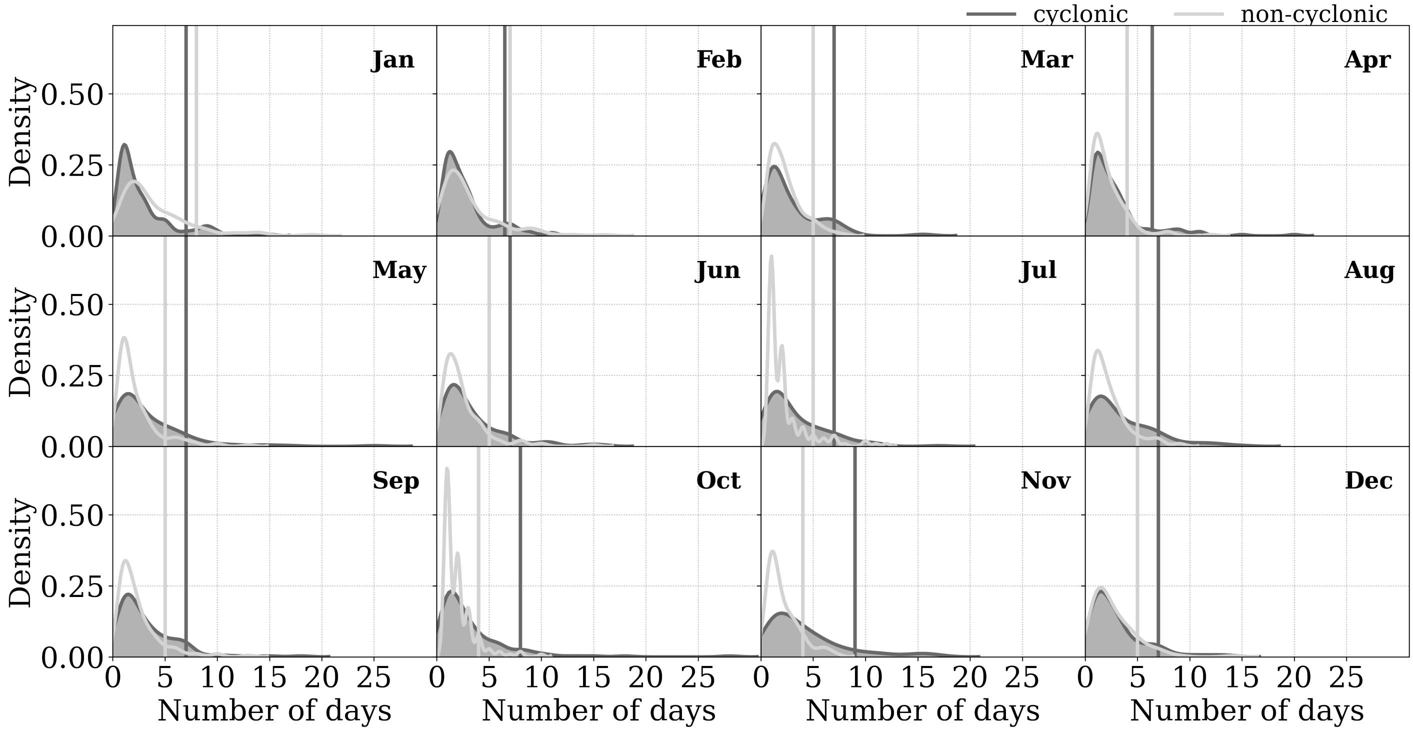


**Figure S1.** Distribution of consecutive days with cyclonic (dark gray) and non-cyclonic (light gray) conditions. The vertical lines represent the $90^{th}$ percentiles.

Long, persistent weather periods are rare at Neumayer. Regardless of the season, weather conditions often change every two to three days. Periods with stable weather conditions for more than 10 days occur in less than 10% of the time.

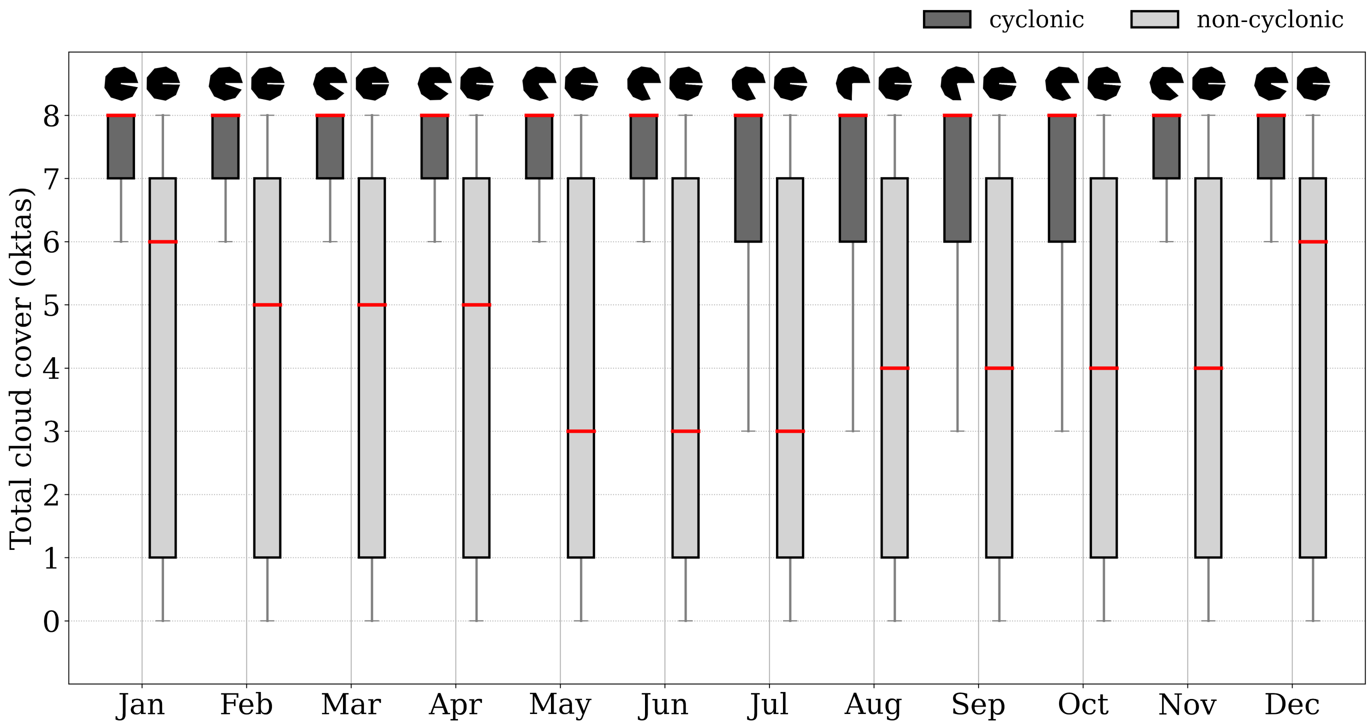


**Figure S2.** Monthly distributions of cloudiness for cyclonic and non-cyclonic conditions. The red lines indicate the respective median, the boxes the inter-quartile range, and the whiskers 1.5 times the inter-quartile range. The SYNOP code has the numbers 1-8 corresponding to 1-8 octas, plus the number 9 meaning "sky not visible". Thus, additional pie plots at the top of the figure show the fraction of available observations.

"Sky not visible" can be due to darkness, fog, or blowing snow, the latter causing the higher fraction of number 9 and thus lower fraction of available data for cyclonic than for non-cyclonic conditions. Under non-cyclonic weather conditions, cloudiness is clearly lower than under cyclonic conditions. The still relatively high median cloudiness in summer is partly due to the transition periods between cyclonic and non-cyclonic weather.

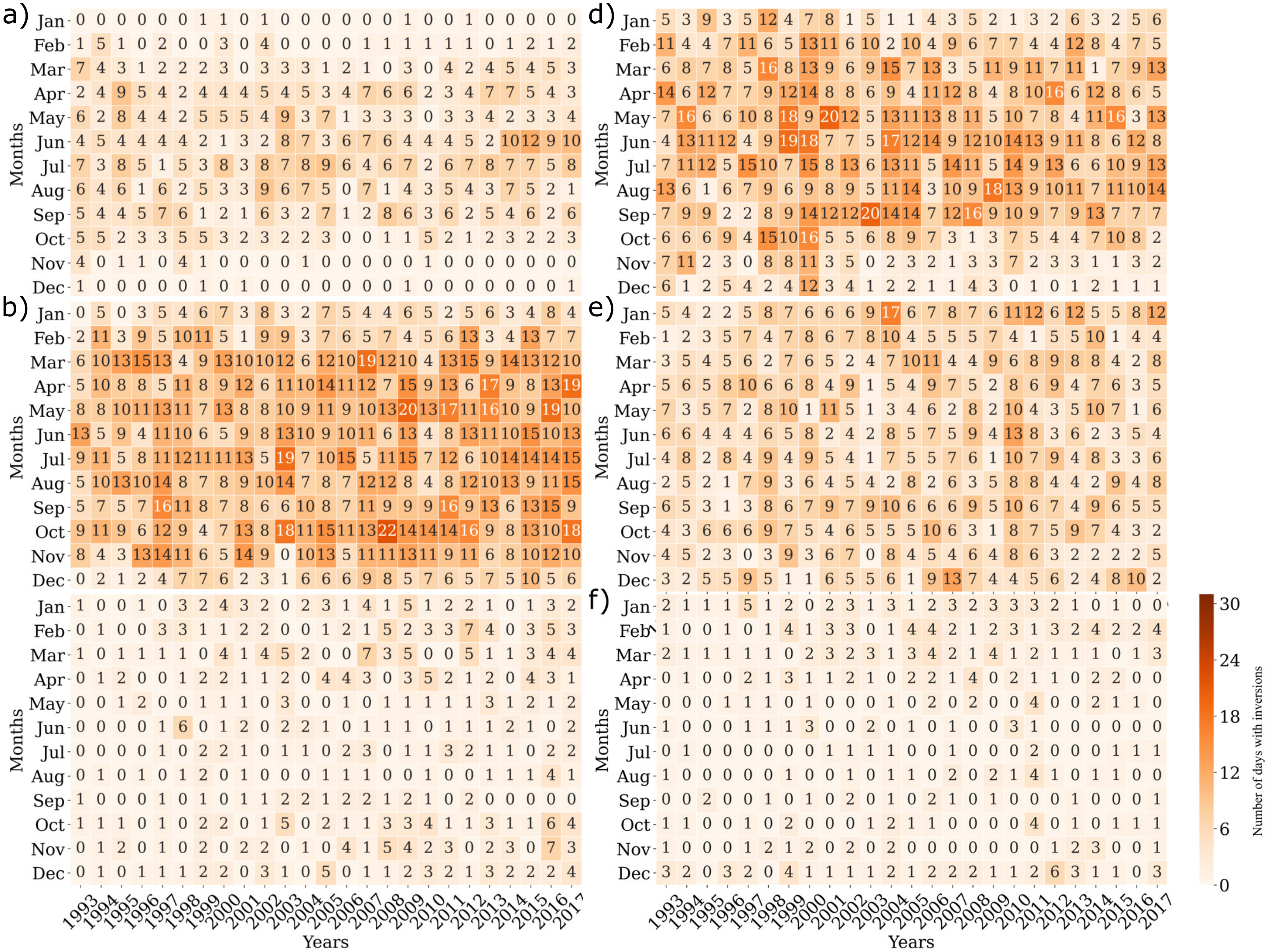


**Figure S3.** Monthly distribution of days with temperature inversions between 1993-2017 at the three different levels (first level: a and d; second level: b and e; third level: c and f) for cyclonic (a, b, c) and non-cyclonic (d, e, f) conditions

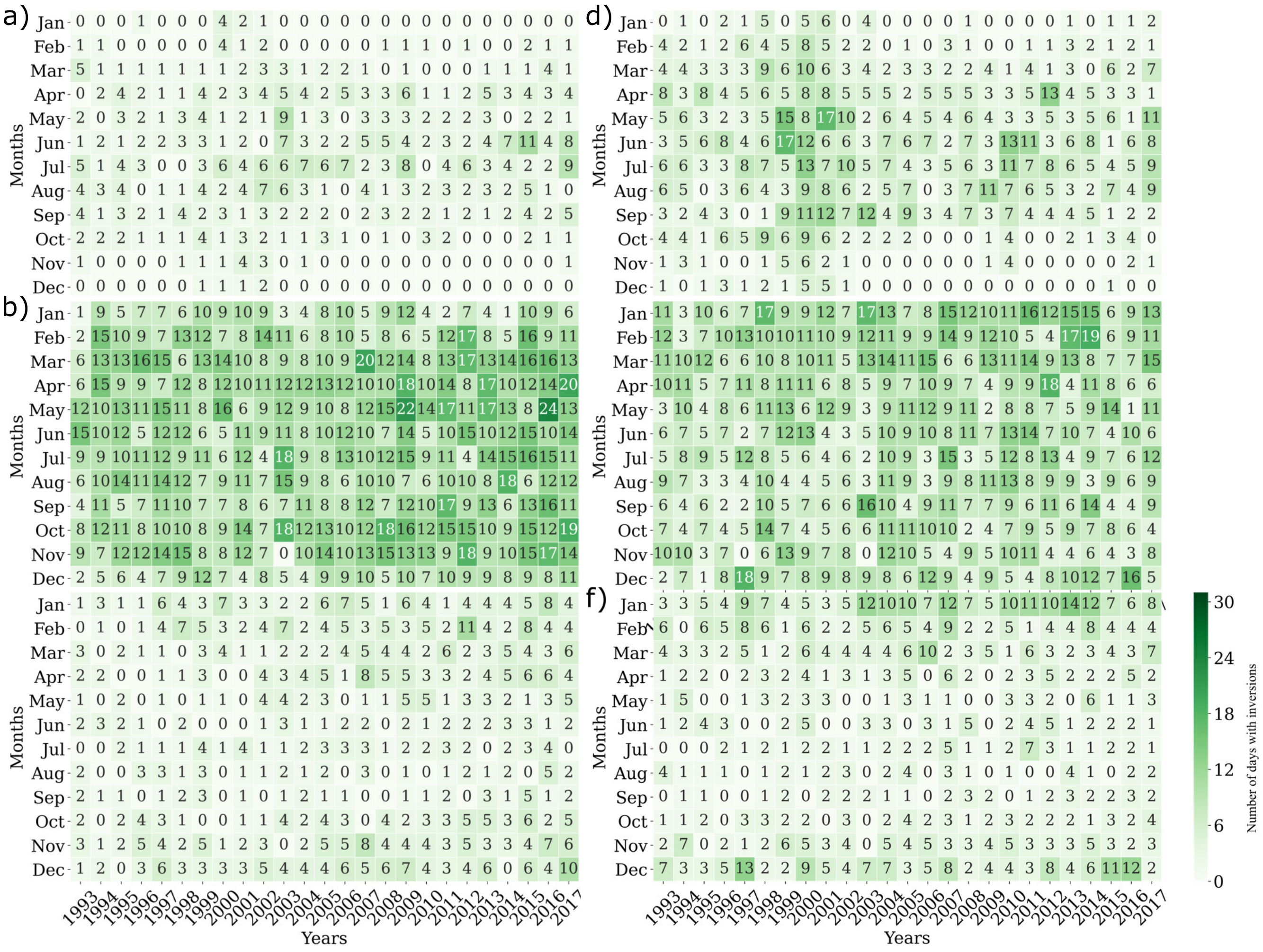

**Figure S4.** Monthly distribution of days with humidity inversions between 1993-2017 at the three different levels (first level: a and d; second level: b and e; third level: c and f) for cyclonic (a, b, c) and non-cyclonic (d, e, f) conditions

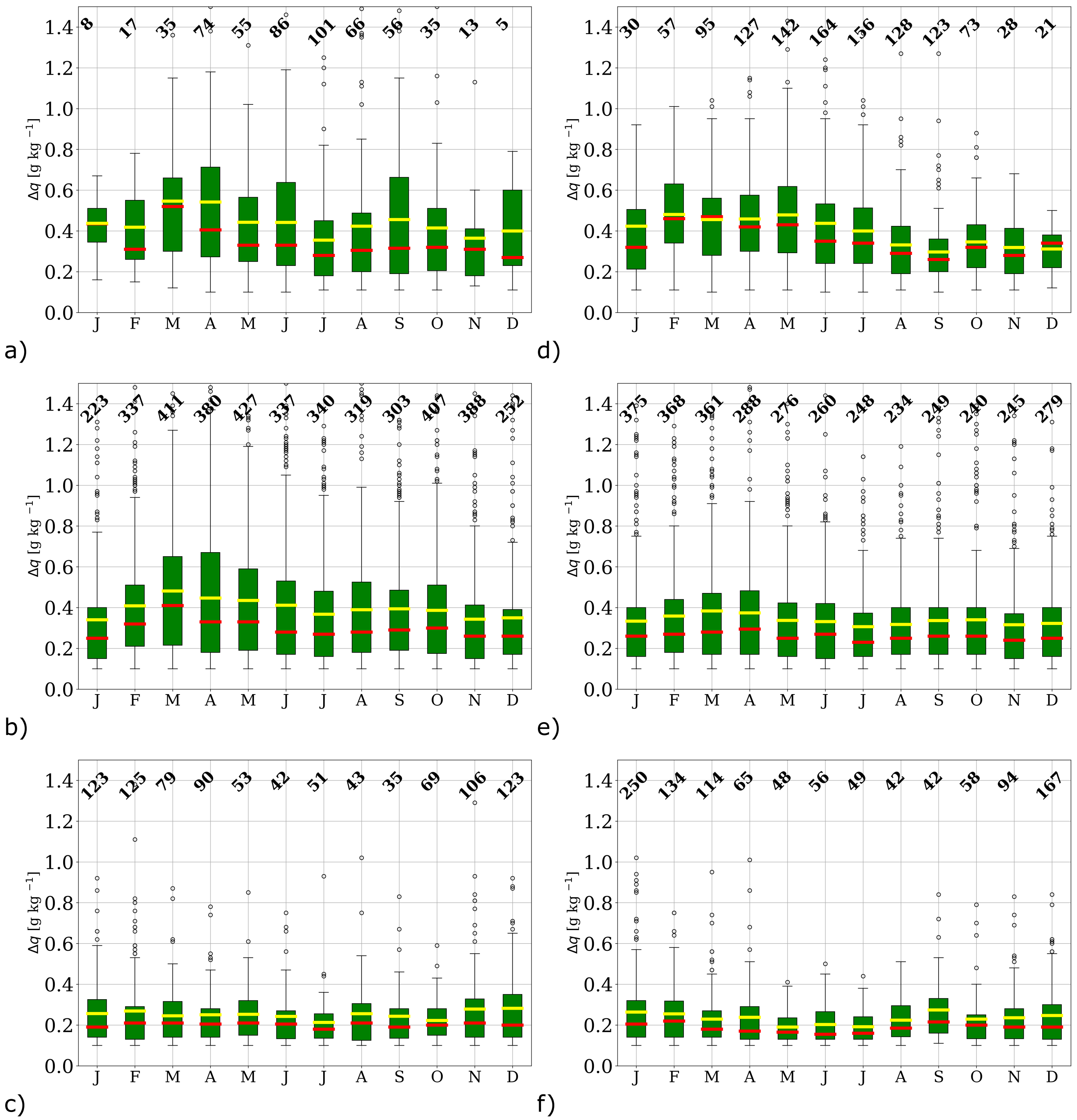


**Figure S5.** Monthly mean (yellow) and median (red) humidity inversion strength at the three different levels (first level: a and d; second level: b and e; third level: c and f) for cyclonic (a, b, c) and non-cyclonic (d, e, f) conditions; numbers at the top of each subplot indicate the monthly sample size. Boxplots are described in Figure S1.

Humidity inversion strength generally shows a very high variability for both cyclonic and non-cyclonic conditions and at all levels. Contrary to temperature inversions, at the surface, cyclonic and non-cyclonic inversions have similar strength. Also, the first and the second level show similar values (approximately between 0.2 and 0.7 g kg$^{-1}$, but the distribution over the year is more even at the second level. Particularly for cyclonic conditions (Fig. S5b) there seems to be a minimum in summer and a relatively clear maximum in fall, whereas for non-cyclonic conditions (Fig. S5e) only a weak maximum in fall and generally much lower variability over the year than at the surface level can be seen. Fall maxima also occur at the surface level, however, variability is so large here that it is not possible to define a distinguished peak. The numerous outliers demonstrate that humidity inversions at the second level can be stronger than at the surface, and they can be equally strong for both weather conditions. Whereas humidity inversions have similar strength for both weather conditions, specific humidity is always higher under cyclonic conditions.

At the third level, similar to temperature inversions, humidity inversions are mostly weak (approximately 0.2 g kg$^{-1}$) and no clear annual cycle or difference between the two weather conditions is found. However, occasionally, even at this high level, inversion strengths similar to those at the surface can occur.

**Table 1.** Statistical significance for temperature inversion features based on the Wilcoxon rank-sum test with the null hypothesis testing whether cyclonic and non-cyclonic conditions are drawn from the same distribution. Dark and light green shading indicate p-values < 0.05 and < 0.1, respectively.

| | | J | F | M | A | M | J | J | A | S | O | N | D |
|---|---|---|---|---|---|---|---|---|---|---|---|---|---|
| dT | 1 | 0.486 | <0.01 | <0.01 | <0.01 | <0.01 | <0.01 | <0.01 | <0.01 | <0.01 | <0.01 | 0.466 | 0.746 |
| | 2 | <0.01 | 0.827 | <0.01 | <0.01 | 0.034 | 0.157 | <0.01 | 0.06 | 0.089 | 0.316 | 0.052 | 0.042 |
| | 3 | 0.356 | 0.041 | 0.179 | 0.388 | 0.244 | 0.675 | 0.475 | 0.194 | 0.824 | 0.765 | 0.861 | 0.316 |
| dZ | 1 | 0.771 | 0.615 | 0.84 | 0.556 | 0.585 | 0.309 | 0.406 | 0.993 | 0.3 | 0.369 | 0.128 | 0.579 |
| | 2 | 0.864 | 0.209 | 0.497 | 0.028 | 0.412 | 0.865 | 0.02 | 0.797 | 0.92 | 0.951 | 0.106 | <0.01 |
| | 3 | 0.416 | 0.418 | 0.548 | 0.06 | 0.337 | 0.401 | 0.868 | 0.987 | 0.597 | 0.576 | 0.654 | 0.939 |
| dT/dZ | 1 | 0.853 | <0.01 | <0.01 | <0.01 | <0.01 | <0.01 | <0.01 | <0.01 | <0.01 | <0.01 | 0.076 | 0.405 |
| | 2 | <0.01 | 0.284 | <0.01 | 0.023 | 0.078 | 0.194 | 0.085 | 0.13 | 0.062 | 0.216 | 0.268 | 0.586 |
| | 3 | 0.928 | <0.01 | 0.575 | 0.881 | 0.828 | 0.903 | 0.259 | 0.261 | 0.695 | 0.483 | 0.867 | 0.281 |

**Table 2.** Statistical significance for humidity inversion features based on the Wilcoxon rank-sum test with the null hypothesis testing whether cyclonic and non-cyclonic conditions are drawn from the same distribution. Dark and light green shading indicate p-values < 0.05 and < 0.1, respectively.

| | | J | F | M | A | M | J | J | A | S | O | N | D |
|---|---|---|---|---|---|---|---|---|---|---|---|---|---|
| dq | 1 | 0.343 | 0.352 | 0.206 | 0.672 | 0.094 | 0.554 | 0.026 | 0.276 | 0.032 | 0.872 | 0.989 | 0.77 |
| | 2 | 0.661 | <0.01 | <0.01 | 0.016 | <0.01 | 0.012 | 0.053 | 0.022 | <0.01 | 0.025 | 0.316 | 0.886 |
| | 3 | 0.583 | 0.561 | 0.398 | 0.32 | 0.015 | 0.366 | 0.515 | 0.823 | 0.208 | 0.649 | 0.305 | 0.358 |
| dZ | 1 | <0.01 | 0.99 | 0.56 | 0.108 | 0.558 | 0.042 | 0.934 | 0.012 | 0.057 | 0.429 | 0.417 | 0.495 |
| | 2 | 0.465 | 0.169 | 0.066 | 0.086 | 0.085 | 0.856 | 0.554 | 0.157 | 0.271 | 0.742 | 0.134 | 0.9 |
| | 3 | 0.582 | 0.077 | <0.01 | 0.674 | <0.01 | 0.092 | 0.408 | 0.047 | 0.248 | 0.563 | 0.193 | 0.014 |
| dq/dZ | 1 | 0.068 | 0.508 | 0.992 | 0.034 | 0.132 | <0.01 | 0.136 | 0.112 | 0.811 | 0.674 | 0.425 | 0.77 |
| | 2 | 0.642 | <0.001 | <0.001 | <0.001 | <0.001 | 0.077 | <0.001 | 0.016 | 0.266 | 0.116 | 0.962 | 0.78 |
| | 3 | 0.802 | 0.092 | <0.001 | 0.38 | <0.001 | 0.079 | 0.263 | 0.08 | 0.959 | 0.642 | 0.03 | <0.001 |

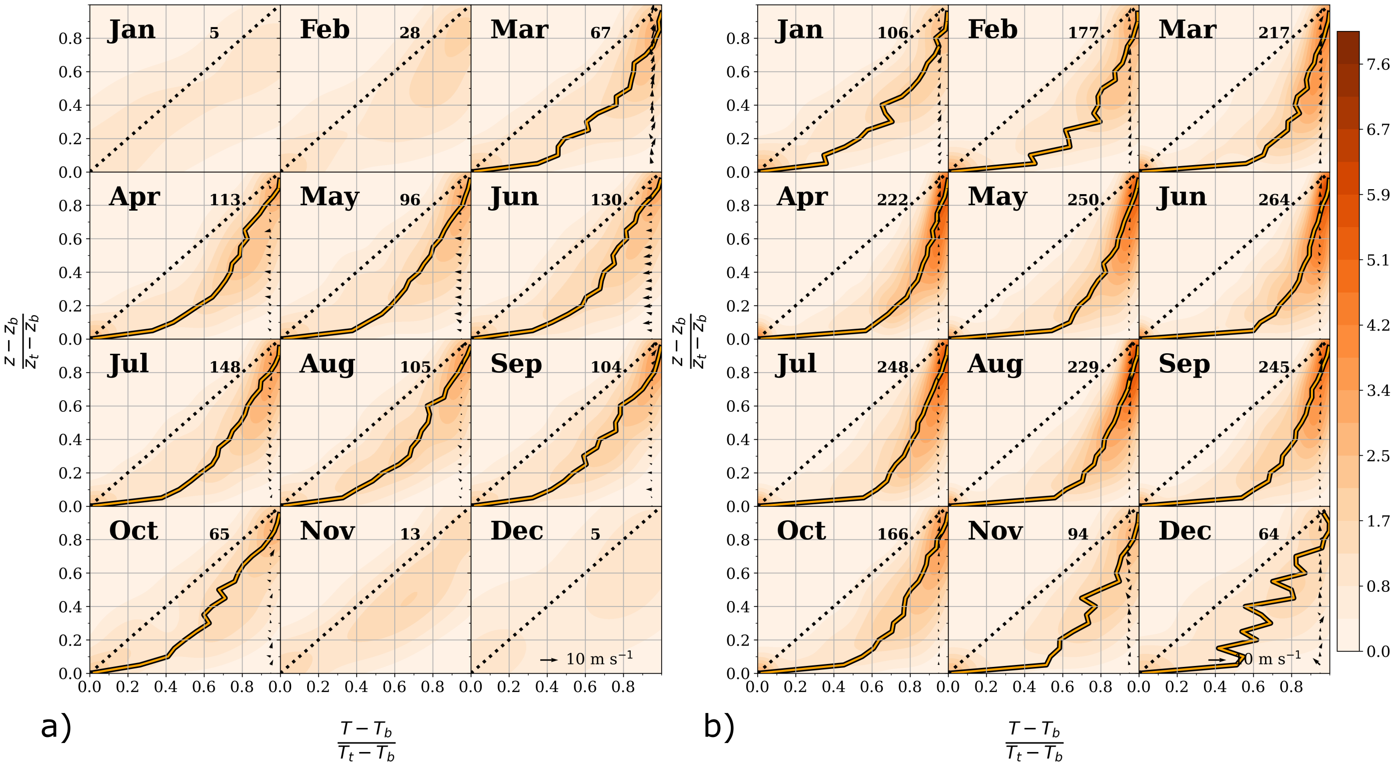


**Figure S6.** 2-dimensional probability density function (PDF) of temperature and height for surface-based inversions under cyclonic conditions (a) and non-cyclonic conditions (b). Temperature and height within the inversion layer were normalized by inversion strength and depth. Additionally, the mean vertical temperature profiles are shown. The normalized profile and the wind vector are vertically averaged in 0.05 bins. The 1:1 line represents a constant temperature increase rate with height within the inversion layer.

Surface-based inversions under cyclonic conditions are relatively rare and according to our definition presumably often represent the transition periods between cyclonic and non-cyclonic conditions as discussed in Section 2.4. Thus, we included this figure for the sake of completeness, but refrain from a detailed discussion here.

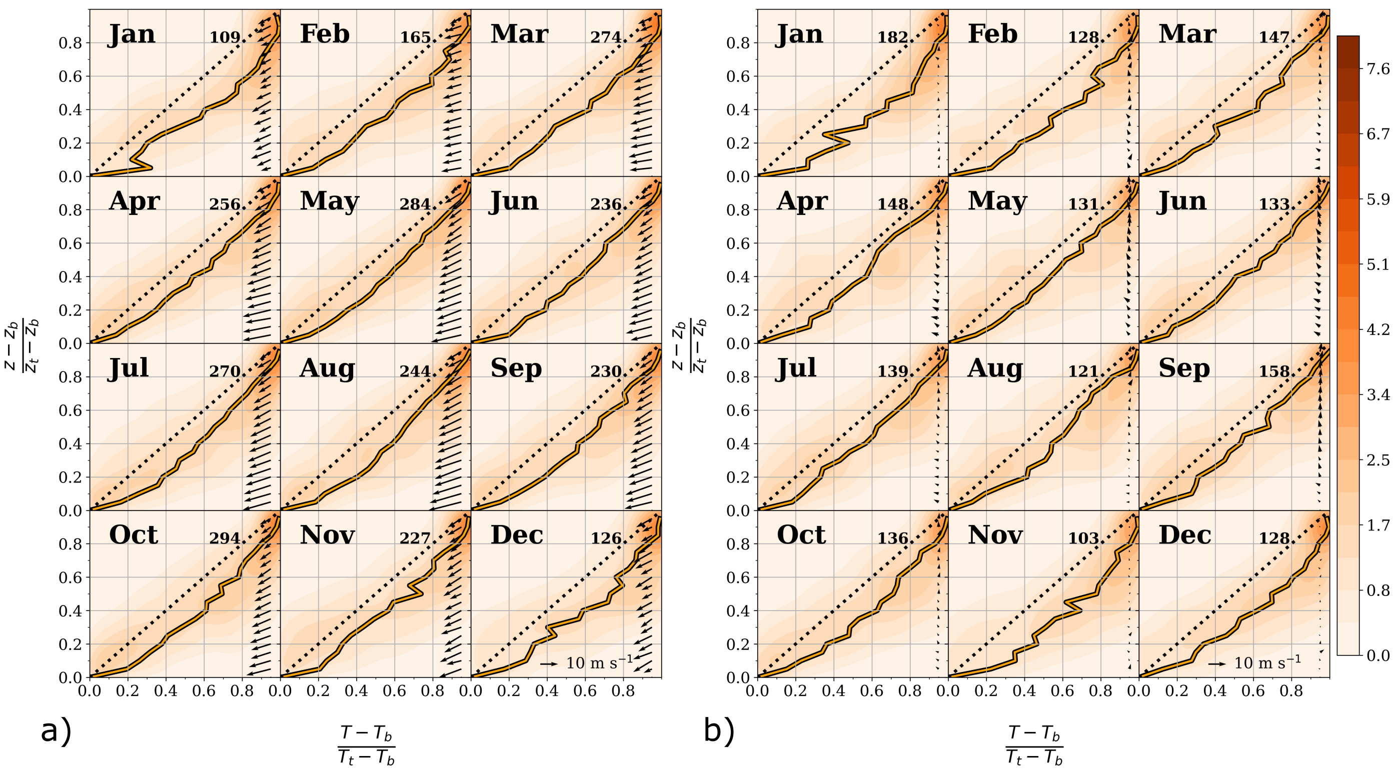


**Figure S7.** 2-dimensional probability density function (PDF) of temperature and height for the second inversion level under cyclonic conditions (a) and non-cyclonic conditions (b). Temperature and height within the inversion layer were normalized by inversion strength and depth. Additionally, the mean vertical temperature profiles are shown. The normalized profile and the wind vector are vertically averaged in 0.05 bins. The 1:1 line represents a constant temperature increase rate with height within the inversion layer.

Under both weather conditions the temperature increase with height is more even than for surface-based inversions, i.e. closer to the 1:1 line. For non-cyclonic conditions winds are generally weak.

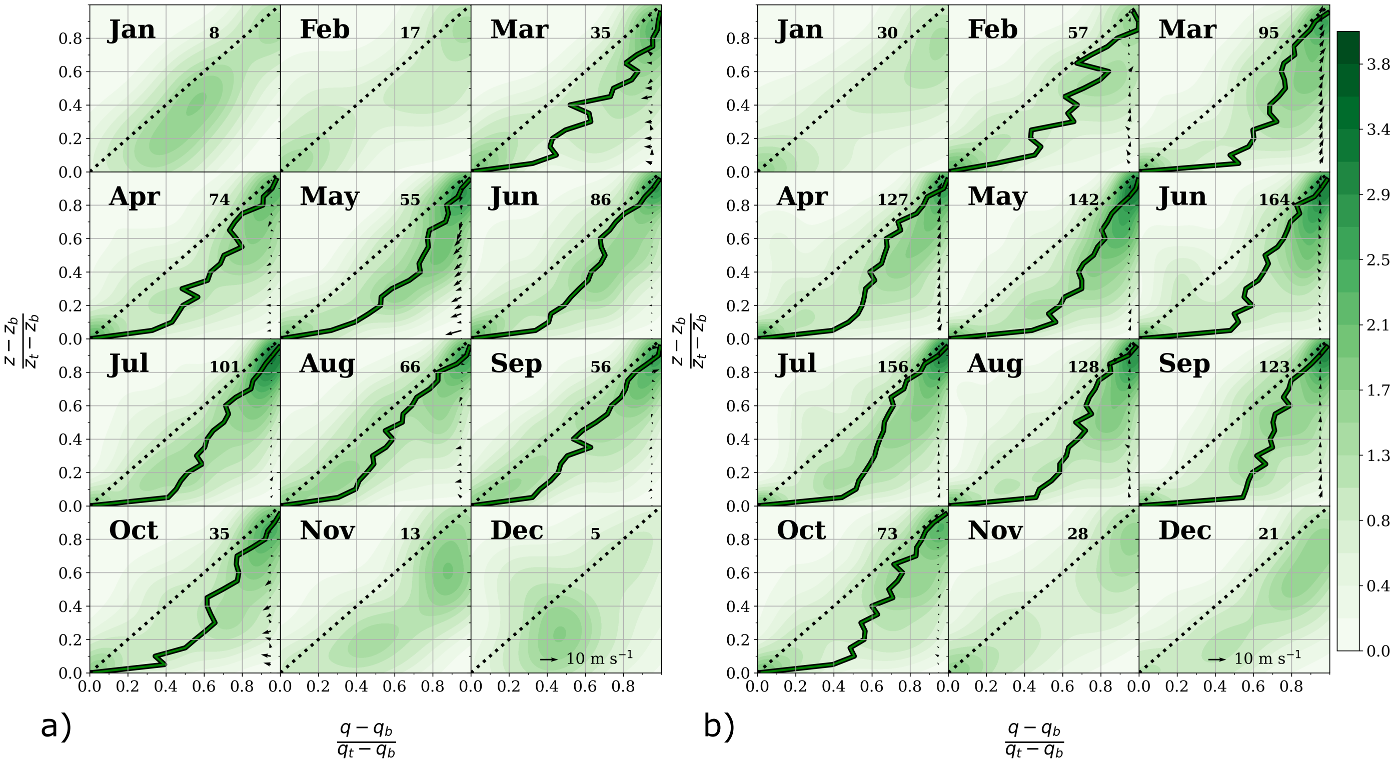


**Figure S8.** 2-dimensional probability density function (PDF) of specific humidity and height for surface-based inversions under cyclonic conditions (a) and non-cyclonic conditions (b). Specific humidity and height within the inversion layer were normalized by inversion strength and depth. Additionally, the mean vertical specific humidity profiles are shown. The normalized profile and the wind vector are vertically averaged in 0.05 bins. The 1:1 line represents a constant specific humidity increase rate with height within the inversion layer.

Similar to surface-based inversions, the strongest increase of humidity is observed in the lowest layer, with up to 60% of the increase in the lowest 20% of the inversion. The variability is generally higher for humidity than for temperature, which leads to less smooth vertical profiles.

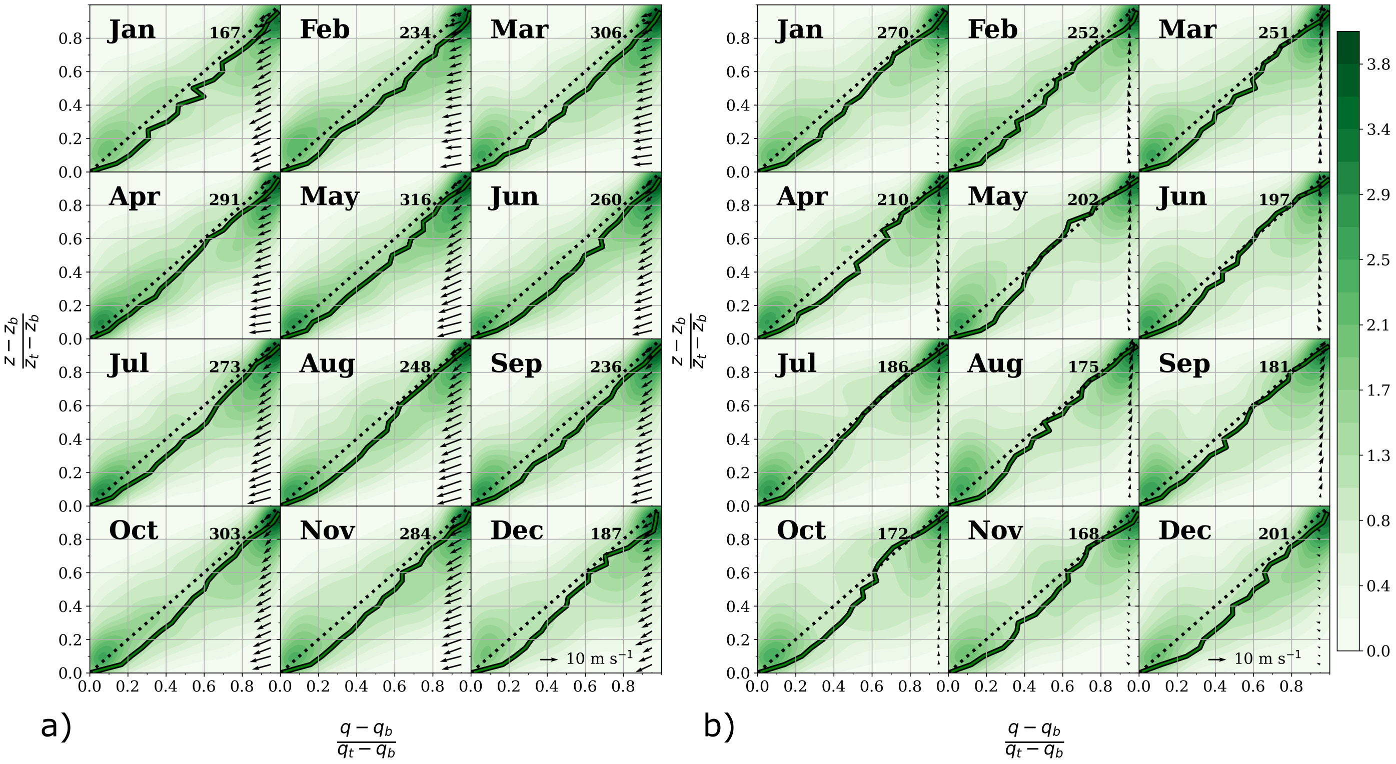


**Figure S9.** 2-dimensional probability density function (PDF) of specific humidity and height for the second inversion level under cyclonic conditions (a) and non-cyclonic conditions (b). Specific humidity and height within the inversion layer were normalized by inversion strength and depth. Additionally, the mean vertical specific humidity profiles are shown. The normalized profile and the wind vector are vertically averaged in 0.05 bins. The 1:1 line represents a constant specific humidity increase rate with height within the inversion layer.

Humidity inversions at the 2nd level exhibit vertical gradients closest to the 1:1 line for both weather conditions, particularly in winter, indicating an almost constant humidity gradient.

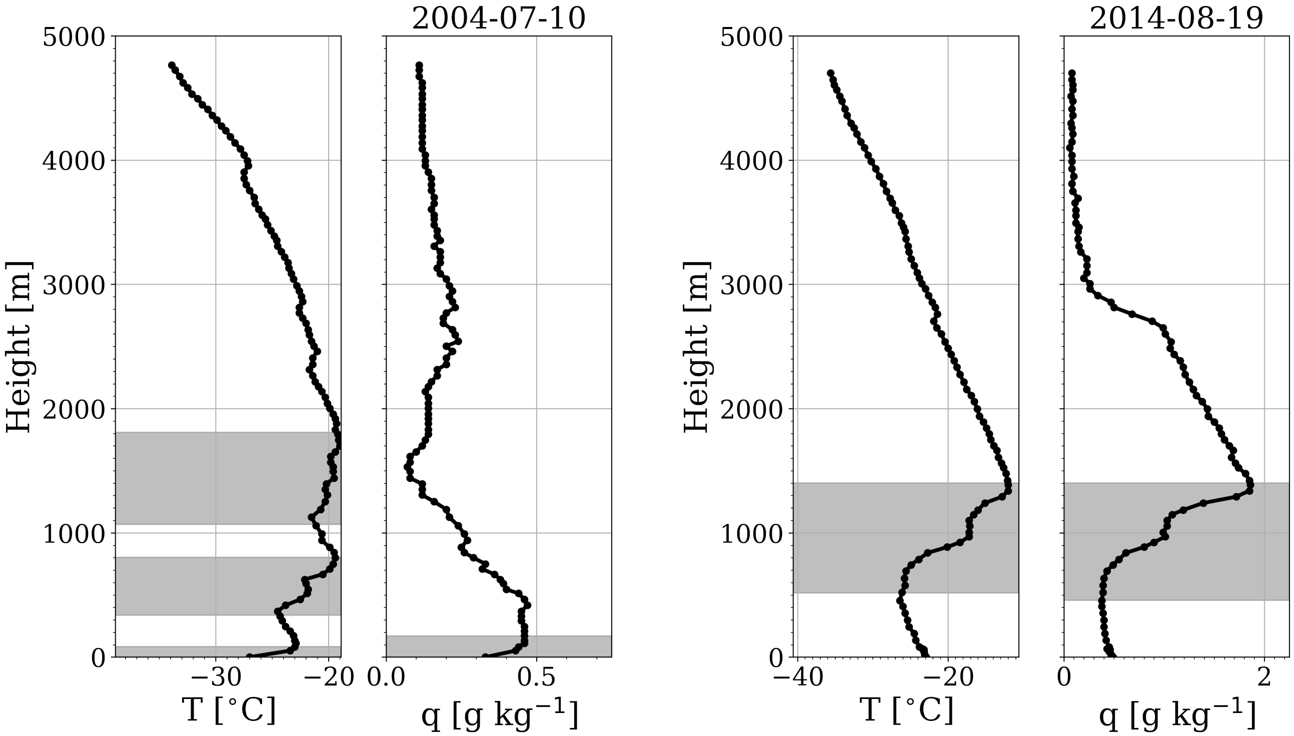


**Figure S10.** Two examples of temperature and specific humidity profiles with inversion layers at different heights. The gray shading indicates the inversion layers identified by the detection algorithm described in Section 2.5 of the manuscript. The vertical profiles are based on raw data from the radiosonde ascents without additional smoothing.
